# The Atypical Protein Kinase C Small Molecule Inhibitor ζ-Stat, and Its Effects on Invasion Through Decreases in PKC-ζ Protein Expression

**DOI:** 10.3389/fonc.2020.00209

**Published:** 2020-02-27

**Authors:** Tracess Smalley, Rainer Metcalf, Rekha Patel, S. M. Anisul Islam, Raja Reddy Bommareddy, Mildred Acevedo-Duncan

**Affiliations:** Department of Chemistry, University of South Florida, Tampa, FL, United States

**Keywords:** PKC-ζ, PKC-zeta, Ect2, clear cell ovarian carcinoma (CCOC), ζ-Stat, zeta-Stat, computational molecular modeling

## Abstract

Ovarian cancer is estimated to reach 22,530 diagnoses and cause 13,980 cancer deaths per year. The most common histology diagnosed of ovarian cancer is epithelial ovarian carcinomas (EOC). An aggressive epithelial subtype is clear cell ovarian carcinoma (CCOC) and is characterized as a non-serous ovarian cancer. Protein kinase C (PKC) is an enzymatic family of proteins that have been found to be a component in cancer progression, tissue invasion, and metastasis. The atypical PKC (aPKC) isoforms, PKC-ι and PKC-ζ, have been suggested to participate in the increased proliferation of ovarian cancers. Previous studies have indicated that novel aPKC inhibitors ICA-1S and ζ-Stat decreased the migratory behaviors of colorectal cancer cells and were selective for PKC-ι/λ and PKC-ζ, respectively. The aims of this investigation were to further determine the binding mechanisms of ζ-Stat, expand on the tissue range of these compounds, investigate the therapeutic potential of ζ-Stat in CCOC, and to illustrate the disruption of invasion via the PKC-ζ signaling cascade. The methods utilized were molecular docking and virtual target screening, Western blot analysis, end-point PCR, GST pull down, cell viability and invasion and migration assays. We discovered that the small molecule inhibitor, ζ-Stat, is a prospective drug candidate to investigate as a novel potential treatment for CCOC. We also found that the PKC-ζ/Ect2/Rac1 activation pathway was decreased by ζ-Stat, which in turn decreased invasive behavior of CCOC.

## Introduction

In 2019, it is estimated that ~1.7 million new incidences of cancer will be diagnosed and 600,000 cancer deaths will occur (1). Cancer has become the leading cause of death in 21 states, despite decreases in cancer death rates since 1991 (2). Specifically, ovarian cancer is estimated to reach 22,530 diagnoses and cause 13,980 cancer deaths per year (1). This constitutes 5% of cancer deaths among women and is responsible for being the most lethal gynecological cancer diagnosis (3).

The most common ovarian cancer diagnosis is epithelial ovarian carcinomas (EOC), which constitutes 85–90% of diagnosis (3). Of this percent, clear cell ovarian carcinoma (CCOC) represents 5% of incidence and presents unique pathological features and a chemo resistant phenotype (4, 5). CCOC is the third most common subtype of ovarian cancer, has a higher risk of reoccurrence, and lower survival rate (4). Furthermore, CCOC is characterized as a non-serous (NS) ovarian cancer and has been found to be more invasive than high grade serous ovarian carcinoma (HGSOC). It has been found that patients with NS tumors have poor prognosis (6). Consequently, CCOC is proposed to be associated with endometriosis and it has been suggested that the pre-cancerous lesions from endometriosis can lead to CCOC (relative risk = 12.4) (7). Endometrial cancer and CCOC have been shown to have an overexpression of Protein kinase C (PKC) isoforms which play important roles in these cancers development and resistance (8).

Protein kinase C (PKC) is an enzymatic family of proteins that have been found to be a component in cancer progression (9). These proteins phosphorylate the serine and threonine residues of substrates and are generally activated by compounds such as diacylglycerol (DAG), calcium (Ca^2+^) and phorbol esters (9). There are three classifications within the PKC family which include the *conventional* PKC-α, βI, βII (splice variant), γ, the *novel* PKC-δ, ε, η, θ, and the *atypical* PKC-ζ, ι/λ (9).

The atypical PKC isoforms, PKC-ι and PKC-ζ, have been suggested to participate in the increased proliferation of ovarian cancer (10). PKC-ι has also been identified as a highly amplified gene in CCOC (4) and is noted for its role in apical-basal polarity loss (10). In addition, due to mutations in the PIK3CA gene and inactivation of Phosphatase and Tensin Homolog (PTEN), the Phosphoinositide 3-Kinase (PI3K)/Serine Threonine Kinase 1 (AKT)/Mechanistic Target Of Rapamycin Kinase (mTOR) pathway has also been upregulated in CCOC (5, 11–13). The upregulation of this pathway increases the expression of downstream survival targets (e.g., PKC-ζ). PKC-ζ has been shown to be involved in tumorigenesis, tissue invasion, and cancer progression through the modulation of cell migration machinery, such as Ras Homolog Family Member A (RhoA), Rac Family Small GTPase 1 (Rac1), and Epithelial Cell Transforming 2 (Ect2) (14–16).

The ECT2 gene is highly amplified in CCOC and may increase migratory behavior (4). Ect2 is a Rho GTPase specific guanine nucleotide exchange factor (GEF) which activates this family of proteins by the addition of a phosphate group to Guanosine diphosphate (GDP) (17). The overexpression of Ect2 protein promotes increased activation of the Rho GTPases, which in turn can facilitates invasion through cytoskeleton reorganization (18).

**Figure 1:**
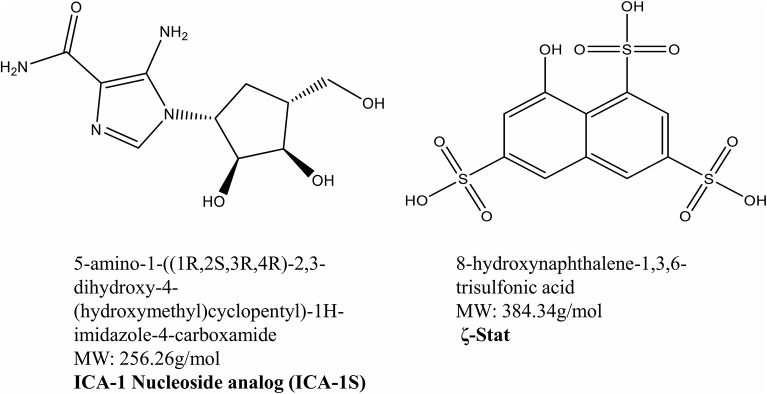
Atypical PKC inhibitors. The molecular structures and molecular weights of ICA-1S and ζ-Stat. ICA-1S was synthesized by United Chemistry Resources and ζ-Stat was distributed by the NCI.

Previous studies have indicated that novel aPKC inhibitors ICA-1S and ζ-Stat (Figure 1) decreased the migratory behaviors of colorectal cancer cells and were selective for PKC-ι/λ and PKC-ζ, respectively (16, 19). These small molecule inhibitors were also shown to decrease cell viability in colorectal cancer and melanoma (16, 19).

Furthermore, computational molecular docking was performed on PKC-ι and a homology model of PKC-ζ (since there is no crystal structure available) with ICA-1S and ζ-Stat (19). In this study, the authors suggested that ICA-1S bound to a potential allosteric pocket (19). However, a more in-depth analysis of ζ-Stat is needed for subsequent studies. The further development of computational modeling is pivotal for drug discovery optimization and helps push these small molecule inhibitors toward a clinical setting. Computational studies can generate mechanistic understandings of the activity these compounds present, can allow for inhibitor improvement, and can institute further signaling investigations.

It has been suggested that the distal downstream signal cascade of PI3K/aPKC pathway should be targeted due to the genotypic and phenotypic reliance of this pathway in CCOC for survival and invasion. The aims of this study were to further determine the binding mechanisms of ζ-Stat, expand on the tissue range of these compounds by investigating the effects in CCOC cell lines, investigate the therapeutic potential of ζ-Stat in CCOC, and to illustrate the disruption of invasion via the PKC-ζ signaling cascade.

## Materials and Methods

### Antibodies and Reagents

The small molecule inhibitors, ζ-Stat and ICA-1S, were obtained from the National Institute of Health (NIH) branch National Cancer Institute (NCI) and United Chem Resources in Birmingham Alabama, respectively. The sources of cell lines, reagents and antibodies were: TOV21G and ES-2 CCOC cell lines (American Type Culture Collection, USA); SHT290 normal endometrial stromal cell line (Kerafast, USA); MCDB 121, Media 199, F12K, penicillin and streptomycin, trypsin, Dulbecco's phosphate buffered saline (DPBS) and Mito + (Corning, USA); McCoy's media (HyClone, USA); Opti-MEM I (Gibco, USA); Fetal bovine serum (FBS, Atlanta Biologicals, USA); human insulin (MP Biomedicals, LLC, France); dimethyl sulfoxide (DMSO, Sigma Aldrich, USA); Water-Soluble Tetrazolium (WST-1, Roche, USA); Halt protease and phosphatase inhibitors cocktail and Protein A/G magnetic beads (Thermo Scientific, USA); anti- PKC-ζ (9372s, 1:1000, Cell Signaling, USA); anti- PKC-ι (610178 1:1000, BD, USA); anti-β-actin (A3854, 1:40000, Sigma Aldrich, USA); anti-RhoA (ab54835, 1:4000, Abcam, USA); anti-Ect2 (07-1364, 1:1000, Millipore, USA); anti-β-tubulin (5346t, 1:1000, Cell Signaling, USA); anti-PARP (9532s, 1:1000, Cell Signaling, USA); Donkey anti-rabbit IgG Alexa-488 (A21206, 1:500, Invitrogen, USA); Goat anti-rabbit (170-6515, 1:2000, Bio-Rad Laboratories, USA); Goat anti-mouse (170-6516, 1:2000, Bio-Rad Laboratories, USA); Activated Rac1 pulldown kit (BK035, Cytoskeleton, USA); 96-well transwell insert and basement membrane extract (BME; both Corning Inc., Corning, NY, USA); RNA bee (Amsbio, United Kingdom); Qiagen RT Kit (205113, Qiagen, Germany); RhoA PCR primers (HP100025, Sino Biological, USA); Glyceraldehyde 3-phosphate dehydrogenase (GAPDH) PCR primers (Eurofins, USA); NE-PER Nuclear and Cytoplasmic Extraction Reagents (78835, Thermo Fisher Scientific, USA).

### Analysis of Somatic Gene Mutations for Ovarian Cancer, CCOC and CCOC Cell Lines

The selection of CCOC cell lines employed the COSMIC database (20). Initially, the search was focused on all ovarian subtypes in the database. The search was then re-focused on clear cell carcinomas. Furthermore, the COSMIC cell line project was utilized for the analysis of mutations in TOV21G and ES-2 cells (20).

### Computational Analysis of aPKCs

#### Protein Preparation

Protein model systems for PKC-ι and PKC-ζ were prepared using the Schrodinger software suite (21). Protein structure coordinates were downloaded from the Protein Data Bank (PDB) (22, 23). The PKC-ι model was generated from the PDB 3A8W entry co-crystallized with adenosine triphosphate (ATP) (24). The apo structure, PDB 38AX, was used to cross reference conformational states from Molecular Dynamics (MD) simulations. No crystal structure currently exists of PKC-ζ, necessitating the need for homology modeling to attempt to produce a potentially viable docking model for PKC-ζ. Two PKC-ζ models were built, one utilizing SWISS-MODEL (25–27) and another employing the Prime homology program (28–30) using the human PKC-ζ sequence (UniProt Q05513) and the crystallized PKC-ι structures as templates. PDB systems were prepared with the Protein Preparation Wizard (PrepWizard) in Maestro (30–34). Cofactors used in crystallization (such as sulfate or phosphate ions), ligands, and additional protein dimers were deleted. Bond orders were then assigned, including disulfide bridges, and original hydrogens were deleted and later replaced to reduce bad contacts and other crystal artifacts before protonation and hydrogen bond optimization. All waters were retained for assisting in the determination of side chain protonation states and initial hydrogen bond optimization. Missing side chains were added and optimized using Prime. Hydrogen atoms were then added to the protein, remaining cofactors, and to any added structural waters. The program PROPKA (35) was used for the prediction of protein ionization states at 7.4 pH and ProtAssign was used for hydrogen bond optimization. After automatic hydrogen assignment, visual inspection was used to flip residues and change protonation states at protein-protein interfaces if and when appropriate.

#### Molecular Dynamics

MD simulations were performed with the Desmond MD program (36–39). A cubic simulation box was created extending at least 10 Å from the protein with imposed periodic boundary conditions. TIP3P waters (40) were added to solvate the simulation box and was then electrically neutralized by introducing sodium ions. The OPLS-3 all-atom force field (41) was then applied to all atoms. The SHAKE algorithm (42) was used to constrain all bonds in the system and the REference System Propagator Algorithm (RESPA) (43) with an integration time step of 2 fs was employed. The Particle Mesh Ewald (PME) algorithm was used to calculate long-range electrostatics with a real-space cutoff of 13 Å. Van der Waals interactions were cutoff at 16 Å. The systems were simulated in an NPT ensemble using the Nose–Hoover temperature coupling scheme (44) at a temperature of 310 K and a constant pressure of 1 atm using the Martyna-Tuckerman-Tobias-Klein (MTTK) barostat (45, 46).

All systems were energy minimized with a truncated newtonian conjugate gradient (TNCG) method (47) followed by multiple restrained minimizations to randomize systems before equilibration and final simulation. Heavy atoms of the protein were held fixed during heating for an initial 12 ps NVT ensemble simulation at 10 K with the Berendsen thermostat (48). This was followed by simulations at 1 atm in the NPT ensemble for 12 ps at 10 K and 24 ps at 310 K. Unrestrained equilibration MD was then performed for 24 ps at 310 K and 1 atm. Finally, unconstrained production MD was performed on PKC-ι and PKC-ζ systems for 250 ns. Energies were recorded every 2 ps and trajectory frames were recorded every 5 ps.

Final system equilibration was determined by the observation of asymptotic behavior of the potential energy, Root Mean Square Deviation (RMSD), and Radius of Gyration (Rg) profiles and visual inspection of trajectories guided by Root Mean Square Fluctuation (RMSF) profiles (Supplementary Material).

#### Consensus Docking

After equilibration was determined, a hierarchical average linkage clustering method based on RMSD was utilized to determine an average representative structure for the equilibrated PKC-ι system. The program PROPKA was then implemented again on the equilibrated structure to test consistency of side chain protontion states at 7.4 pH. The representative structure was then used for consensus docking incorporating five diverse and complimentary docking methods described below. By applying these varied energy scoring methods, the weaknesses of each method can be identified for a particular model and error statistically minimized, yielding a more accurate summary of ligand binding dispositions and affinities.

As a check for the placement of the grids used in the docking studies and for further analysis of the binding cavities for the ATP binding site and the potential allosteric site, Schrödinger's SiteMap program (49–51) was employed. SiteMap searches the protein structure for likely binding sites and highlights regions within the binding site suitable for occupancy by hydrophobic groups, hydrogen-bond donors, acceptors, or metal-binding functionality of the ligand.

The ligands ICA-1S, ζ-Stat, and ATP were prepared using the program LigPrep (52) and the OPLS-3 all-atom force field was applied to all ligand atoms.

#### Rigid Receptor Docking (RRD)

Rigid docking simulations were performed by Glide (53–55). Glide uses a GlideScore fitness function based on Chemscore (56, 57) for estimating binding affinity, but includes a steric-clash term, adds buried polar terms to penalize electrostatic mismatches, and modifies other secondary terms. Docking simulations used both the standard precision (SP) and extra precision (XP) methods. XP mode is a refinement algorithm enforced only on good ligand poses. Sampling is based on an anchor and refined growth strategy and the scoring function includes a more complete treatment of some of the SP energetic terms, such as the solvation and hydrophobic terms. Docking grids were defined by a rectangular ligand atom inclusion outer box of 22 Å and ligand centroid constraint inner box of 10 Å in the x, y, and z directions originating from the binding cavity centroid defined by SiteMap for the proposed allosteric site and by the original co-crystallized ATP ligand centroid for the ATP binding site.

### Induced Fit Docking (IFD)

The IFD methodology (30, 55, 58–60) incorporates both the docking program Glide to account for ligand flexibility and the Refinement module in the Prime program to account for receptor flexibility. The Schrödinger IFD protocol attempts to model induced-fit effects from alterations in binding site conformation due to ligand binding in order to increase accuracy of binding affinity estimates and prediction of possible binding modes.

The position of the cubic docking grid for the ATP binding site was centered on the original co-crystallized ligand centroid and from the binding cavity centroid defined by SiteMap for the proposed allosteric site with a box size of 29 Å for both. A constrained minimization of the receptor was performed with an RMSD cutoff of 0.18 Å. An initial softened potential Glide docking of the ligand set was then implemented with the standard precision (SP) mode and a van der Waals scaling factor of 0.5 was applied to the non-polar atoms of the receptor and ligands. The resulting top 20 poses of the ligands were used to sample protein plasticity by conformational searches and minimizations of binding pocket residues within 6 Å of any ligand pose for all complexes obtained. The new receptor conformations were then redocked using complexes within 30 kcal/mol from the best scoring structure. Glide docking parameters for this step were reset to the default hard potential function with a van der Waals scaling of 1.0 and SP mode.

The estimated binding affinity of each complex was reported in the GlideScore and used to compare differences between each ligand while the Emodel score is used to inter-compare poses of the ligands. Emodel places more significance on weighting force field components (electrostatic and van der Waals energies), making it better for comparing conformers as opposed to comparing chemically-distinct species.

#### Quantum Polarized Ligand Docking (QPLD)

To account for ligand polarization upon binding, Quantum Mechanics/Molecular Mechanics (QM/MM) docking was performed by the Schrödinger QM-Polarized Ligand Docking Protocol (QPLD) (61–64). The protocol first employs RRD using Glide in SP mode. In this step, the top five poses of each ligand in the initial RRD were used. Potential ligand polarization induced by the protein were then calculated with QSite (63, 65, 66) at the B3LYP/6-31G^*^ level. The ligand force fields were then reconstructed with QM/MM modified charges, redocked, and five poses of each ligand were saved for evaluation.

#### Molecular Mechanics and Generalized Born Surface Area (MM/GBSA)

The MM/GBSA method combines molecular mechanics energy terms and implicit solvation models to calculate the binding-free energy based on docking complexes. The protocol, implemented by the Prime MM-GBSA module, calculates optimized free energies for the free protein and free ligand and references them with the original bound complex energy (67). Polar contributions are calculated using the Generalized Born (GB) model (68), an implicit solvent model is based on a variable dielectric surface Generalized Born (VD-SGB) approach, where the variable dielectric value for each residue was fit to a large number of side-chain and loop predictions while the non-polar energy is estimated using the solvent accessible surface area (SASA) (69). The simulation was performed based on receptor–ligand complex structures obtained from induced fit docking. The obtained ligand poses were minimized using the local optimization feature in Prime, whereas the energies of complex were calculated with the OPLS-3 force field and Generalized-Born/Surface Area continuum solvent model (70). During the simulation process, the ligand strain energy is also considered. A known issue with MM/GBSA is that scores do not accurately reproduce absolute physical binding affinities but display great efficacy at ranking compounds in a relative manner (71–74). We developed a correlation function using a single-layer logistic regression to rescale MM/GBSA scores based on the other docking score algorithms. This retains the ranking accuracy of MM/GBSA and allows us to proportion the results in a minimally biased and physically relevant manner.

#### Virtual Target Screening (VTS)

VTS is a system designed to virtually screen a molecule of interest to a large library of protein structures. The current protein library consists of 1,451 structures with a concentration of kinases. The system is calibrated with a set of small drug-like molecules are docked against each structure in the protein library to produce benchmark statistics. VTS was employed as a theoretical assay of potential kinase activity and gauge of potential biological promiscuity. The calibration procedure allows the analysis to accurately predict inhibitor–kinase binding affinities when Kd <10 μM (defining a hit) and Kd ≥ 10 μM are both considered (72% accuracy in the best case) (75). Therefore, the VTS system is able to robustly discriminate protein binders from non-binders and give some inclination as to potential binding promiscuity of the molecule of interest with respect to the protein group tested.

### Cell Culture

The CCOC cells lines TOV21G and ES-2 were cultured in MCDB 131: Media 199(1:1 ratio) and McCoy's medium, respectively, supplemented with 10% FBS, 100 units/mL of penicillin, and 100 μg/mL of streptomycin. The immortalized normal human endometrial stromal cell line, SHT290, was maintained in F12K: Media 199 (1:1 ratio) and supplemented with 5% FBS, 0.1% Mito+, 2 μg/mL of human insulin, 100 units/mL of penicillin, and 100 μg/mL of streptomycin. All cell cultures represented were passaged <10 times. Cell cultures were maintained in an incubator at 37°C and 5% CO_2_ atmosphere.

### Atypical PKC Expression During Rapid Growth and Cell Cycle Arrested

SHT290, TOV21G, and ES-2 cells were seeded into 100 mm plates and grown to 50% confluency. Rapidly growing cells were harvested at 50% confluence and the cell cycle arrested cells were serum starved for an additional 48 h in reduced serum medium Opti-MEM I (*N* = 3).

### Preliminary Screening of TOV21G and ES-2 With ICA-1S and ζ-Stat

TOV21G and ES-2 cells were seeded into 100 mm plates and grown to 50% confluency. Control cells were treated with equal amounts of DMSO (vehicle) and treatment cells were treated with 3 μM concentrations of ICA-1S and ζ-Stat for 24, 48, and 72 h (*N* = 3). Cells were collected at 24, 48 and 72 h and run on a Western blot.

### Cell Viability Assay

Cells were seeded in to 96-well plate at 800 cells per well with 200 μL of media. Cells were treated with different concentrations of DMSO (vehicle to match treatment, *N* = *12*) and ζ-Stat (1, 3, 5, and 10 μM, *N* = 3). After 72 h of treatment, the cell viability was analyzed using WST-1 at wavelengths 450 and 630 nm. The plates were read on a BioTek SynergyHT microplate reader. Standard curves for each cell line was generated based on the number of cells added and the absorbance recorded.

### Cell Lysate Collection

Media was extracted from the vessel and 250 μL of lysis buffer [Pierce® Immuno Precipitation Lysis Buffer, 25 mM Tris-HCl pH 7.4, 150 mM NaCl, 1 mM EDTA, 1% NP-40 and 5% glycerol] with protease and phosphatase inhibitors was added to the plates. Cells were scraped and collected from the vessel (on ice) and the suspension was sonicated for 3 × 5 s cycles on ice. The samples were centrifuged at 4°C at 12,000 × g for 15 ms. The supernatant (cell lysate) was removed from the cellular membrane pellet and placed in a secondary micro centrifuge tube. Protein content was measured per Bradford Assay Reagent on a BioTek SynergyHT microplate reader at 595 nm.

### Western Blot Analysis

Cell lysates containing equal amounts of protein (20–40 μg) were loaded in each lane and run on sodium dodecyl sulfate-polyacrylamide gel electrophoresis (SDS-PAGE) and transblotted to a 0.45 μm nitrocellulose membrane. Cell lysates were probed with the primary antibodies against PKC-ζ, PKC-ι, RhoA, and β-actin (for loading control) and re-probed with secondary antibodies for development. Immunoreacted bands were visualized by enhanced chemiluminescence per the manufacturer's instructions [Thermo Scientific™ SuperSignal™ West Pico PLUS Chemiluminescent Substrate]. Band densitometry was performed with ImageJ FIJI (76) software and normalized densities were derived by the ratio of the protein of interest over the control (β-actin).

### Endpoint PCR

The mRNA from TOV21G and ES-2 cells treated with vehicle control and 3 μM of ζ-Stat was isolated via the manufacture's protocol for RNAbee. The mRNA was then quantified using the BioTek Synergy HT Take5 plate and cDNA was synthesized using the Qiagen reverse transcription (RT) kit per the manufacturer's protocol. Endpoint polymerase chain reaction (PCR) was performed with primers for RhoA and the house keeping gene GAPDH (forward 5′CCA-CCC-ATG-GCA-AAT-TCC-ATG-GCA-3′ and reverse 5′TCT-AGA-CGG-CAG-GTC-AGG-TCC-ACC-3′). PCR products were analyzed on an agarose gel (*N* = 3).

### Rac1 Activation Assay

Cells were cultured in 150 mm plates and lysed as previously described. The cells were treated with DMSO (control) and 10 μM of ζ-Stat for 72 h and harvested (*N* = 4). Glutathione S-transferase (GST) was tagged to the protein binding domain (PBD) of p21 activated kinase (PAK). A positive control and negative control were performed to determine assay efficiency. Briefly, 500 μg of protein were balanced in 200 μL of cell lysis for each sample. The positive control received 200 μM of non-hydrolyzable guanosine 5′-O-[gamma-thio] triphosphate (GTPγS) and the negative control received 200 μM of guanosine diphosphate (GDP). These samples were incubated at room temperature (RT) for 15 ms. All samples (positive, negative, DMSO control and treatment) were incubated with GST-tagged PAK-PBD agarose beads for 1 h 4°C. These samples were pelleted at 5,000 × g (at 4°C) and washed with Wash Buffer. The pelleted beads were re-suspended with 20 μL of 2X Laemmli sample buffer and boiled at 95°C for 2 ms.

### Preparation of Cytoplasmic and Nuclear Extracts

TOV21G and ES-2 cells were seeded in 100 mm tissue culture plates (1.5 × 10^5^). Cells were treated for 72 h with 10 μM ζ-Stat (DMSO control) and harvested with trypsin. The instructions provided by the manufacturer were followed to fractionate the cytoplasmic and nuclear portions. The extracts were analyzed via immunoblots and translocation of Ect2 was investigated.

### Fluorescent Microscopy

TOV21G cells were seeded into 4 chambered slides at a 500 cells per well concentration and after 24 h, were treated with a vehicle control (DMSO) and 10 μM of ζ-Stat every 24 h for 72 h. Cells were then fixed with 4% paraformaldehyde for 15 ms and immunostained with Ect2 antibody at 4°C overnight with light agitation. The slides were incubated with Alexa 488 rabbit secondary antibody for 1 h at room temperature RT. Subsequently, the slides were stained with Phalloidin conjugated to Texas red dye for 30 ms at RT, mounted with solution containing 4′,6-diamidino-2-phenylindole (DAPI) and imaged on an Olympus BX53 Digital Upright Fluorescent Microscope.

### Invasion Assay

For the evaluation of invasion, cells were serum starved for 48 h, followed by detachment and plating into the upper chamber of a 96-well (8 μm) transwell permeable support, coated with 0.1X BME. Serum (10%) containing media was loaded into the lower chamber as a chemoattractant. Subsequently, TOV21G cells at the upper chamber were treated with 10 μM of ζ-stat for 24 h (*N* = 4). Two experimental treatment groups for the cells were performed: Control (DMSO vehicle) and treatment. The invasive cells that passed into the lower chamber were then fixed with 4% paraformaldehyde, stained with 2% crystal violet in 2% ethanol, washed with distilled water and photographs were captured after drying using a light microscope Motic AE31E. For migration, a similar protocol was followed except without coating the transwell insert with BME. The assay was quantified with ImageJ FIJI software.

### ζ-Stat *in-vivo*

The following experiments outline the investigations of ζ-Stat in TOV21G clear cell carcinoma ovarian xenografts. We have an Institutional Animal Care and Use Committee (IACUC) approved by Adrienne Booker for the discussed studies. The study involved 12 athymic female nude mice weighing between 20–25 g and >10 weeks of age. The 12 mice were divided into two groups after TOV21G cells were implanted (1 × 10^6^ cells/per mouse flank in 0.2 mL of media). The first group was the vehicle control group (*N* = 6), which received 100 μL of 1x DPBS. The second group (*N* = 6) was injected with 100 μL of 20 mg/kg of ζ-Stat dissolved in 1x DPBS. The tumor volume was calculated using the formula: length × width × width × ½. Three days after the implantation of the cells, tumors were treated as of day 0. The treatments were administered every other day subcutaneously intra-tumor and around the tumor site for 35 days.

At the end point of the experiment, tumors and heart serum were harvested. Tumors were imaged and measured, and blood serum was analyzed for enzymatic levels of glucose (GLU), blood urea nitrogen (BUN), alanine aminotransferase (ALT), aspartate aminotransferase (AST), and alkaline phosphatase (ALKP) at the Moffitt Research Facility. Briefly, blood chemistry analysis was performed by initially collecting whole blood in a serum separator tube, which then sat for 20 ms at RT before centrifugation. Once the blood was centrifuged the serum was separated and placed in a specialized sample cup made for the IDEXX CatalystDx. The cup containing the serum and the desired chemistry slides were then placed into the CatalystDx for analysis.

### *In-vivo* Tumor Fractionation

Tumors were re-suspended and sonicated in 700 μL of homogenization buffer [Pierce® Immuno Precipitation Lysis Buffer, 25 mM Tris-HCl pH 7.4, 150 mM NaCl, 1 mM EDTA, 1% NP-40 and 5% glycerol]. The suspension was sonicated for 3 × 5 s cycles on ice. The tumor suspensions were centrifuged at 12,000 × g for 15 min to obtain cell extracts. The supernatant was removed and protein content was measured using Bradford reagents.

### Statistical Analysis

R studio software was used for statistical analyses. A one-way ANOVA with a Tukey's multiple comparisons test was performed for Western Blot analyses and cell viability. A two-tailed unpaired student *T*-test was utilized for the statistical significance of the particle counts for cell migration and invasion, day to day tumor volume, mouse body weight, and individual enzyme levels. The Pearson's Correlation Coefficient (PCC) was utilized for co-localization and was analyzed using ImageJ FIJI software, using the Coloc2 plugin.

## Results

### PIK3CA and ARID1A Are in the Top Mutated Genes in All Ovarian Tissue Types and in CCOC

To understand the genetic landscape of ovarian cancer, we utilized the Catalogue of Somatic Mutations in Cancer bioinformatics database (COSMIC) (20). The results demonstrated that the top mutations in ovarian cancer overall are TP53 (p53), FOXL2 (Forkhead box protein L2), KRAS (Kirsten Ras oncogene homolog), PIK3CA (Phosphatidylinositol 4,5-bispohsphate 3-kinase catalytic subunit alpha), ARID1A (AT-rich interaction domain 1A), and BRAF (B-Raf proto-oncogene) (Table 1). The search was then refocused to only contain CCOC samples and the top two mutated genes found were PIK3CA (33%) and ARID1A (50%) (Table 2). These results suggest that one of the most common gene mutations in ovarian cancer and CCOC is PIK3CA, which is ~10% of mutated samples in all the ovarian tissue in the database. Due to this, the downstream survival targets PKC-ζ and PKC-ι are likely to be overexpressed, amending the need for their explicit targeting.

**Table 1:** Six most common gene mutations in all ovarian cancers.

**Gene**	**Protein Product**	**Protein Function**	**Chromosomal Location (human)**	**Percent Mutation of Samples Tested**	**Highest Percent Mutation**	**Mutation Type**
**Somatic mutations in all ovarian tissue types**						
TP53	p53	Tumor suppressor, regulates cell cycle	17p13.1	46	55.89, 20.23	Substitution missense, other
FOXL2	Forkhead box protein L2	Transcription factor	3q23	20	100	Substitution missense
KRAS	Kirsten Ras oncogene homolog (KRAS proto-oncogene, GTPase)	Regulation of cell division	12p12.1	12	100	Substitution missense
PIK3CA	Phosphatidylinosital-4,5-bisphosphate 3-kinase catalytic subunit alpha	Phosphorylates certain signaling molecules	3q26.3	10	96.88	Substitution missense
ARID1A	AT-rich interaction domain 1A	Regulate transcription by altering chromatin structure	1p35.3	9	40, 38.26, 23.48	Substitution missense, deletion frame shift, insertion frame shift
BRAF	B-Raf proto-oncogene, serine/threonine kinase	This protein plays a role in regulating the MAP kinase/ERKs signaling pathway, which affects cell division, differentiation, and secretion.	7q34	7	97.63	Substitution missense

*The table below describes the six most common gene mutations in all ovarian cancers according to the Catalogue of Somatic Mutations in Cancer database (COSMIC). The gene name, protein product, general function, chromosomal location, percent of all samples with mutation, highest type of mutation and most common mutation type are listed*.

**Table 2:** Somatic mutations in CCOC and CCOC cell lines.

	**Somatic mutations in CCOC**	**Somatic mutations in cell lines**	
**Gene**	**Percent mutation of samples tested**	**TOV21G**	**ES-2**
ARID1A	50	ARID1A^Y551fs*72, *Q*758fs*75^	TP53^S241F^
PIK3CA	33	PIK3CA^H1047Y^	PIK3CA
TERT	17	KRAS^G13C^	
TP53	11	PTEN^K267fs*9, *G*143fs*4^	
KRAS	8		

*The table below describes the five most common gene mutations in CCOC according to the Catalogue of Somatic Mutations in Cancer database (COSMIC). The gene name and percent of samples with mutation are listed. The gene mutations for TOV21G CCOC cells and ES-2 CCOC cells are listed with specific mutation type*.

In addition, the PIK3CA mutation was used to select two cell lines that would be representative of this mutation in CCOC. Two commonly utilized cell lines, TOV21G and ES-2 were selected based on their genetic profile. While both cell lines possess a PIK3CA mutation, TOV21G has an ARID1A mutation and ES-2 has a TP53 mutation (Table 2).

### Select Inhibitor Effects on PKC-ζ and PKC-ι Protein Expression

**Figure 2:**
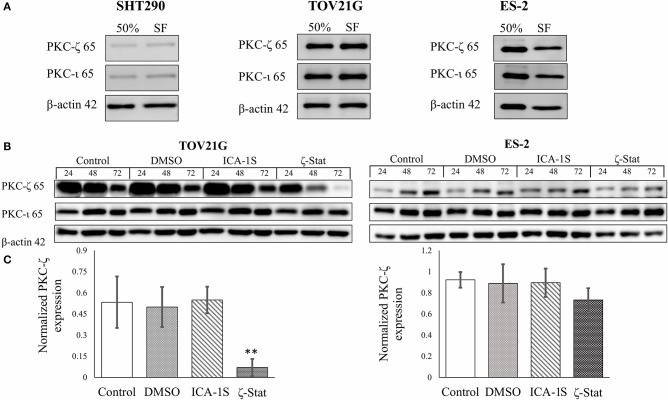
PKC-ζ and PKC-ι protein expression in rapidly growing and serum starved cells and the effects of ICA-1S and ζ-Stat on PKC-ζ and PKC-ι protein expression in SHT290, TOV21G, and ES-2 CCOC cell lines. **(A)** SHT290, TOV21G, and ES-2 cell lines were harvested at 50% and 48 h after serum starvation (SF) (*N* = 3). The membranes were probed with anti-PKC-ζ and PKC-ι to investigate protein expression. **(B)** TOV21G and ES-2 cell lines were treated with 3μM ζ-Stat for 24, 48 and 72 h. An untreated control and vehicle control (DMSO) are also illustrated. The immunoblots were probed with PKC-ζ, PKC-ζ, and β-actin (loading control) (*N* = 3). **(C)** The raw data's densitometry was quantified using ImageJ software and analyzed with a one-way ANOVA. Standard deviation is represented (**p < 0.01).

To determine which inhibitors affect PKC-ζ and PKC-ι protein expression in CCOC, Western Blots were employed with ICA-1S and ζ-Stat (Figure 1) as potential inhibitors. Initially, the expression of these aPKCs were investigated in rapidly growing cells (50%) and cell cycle arrested (serum free, SF) (Figure 2A). The density of each band was quantified using analytical software. The results showed that ICA-1S did not affect PKC-ζ or PKC-ι protein expression (Figure 2B); however, ζ-Stat substantially decreased the expression of PKC-ζ in TOV21G cells (*p*-value 0.00225, F = 9.5709, *t* = −4.413) and ES-2 cells (not significant) but not PKC-ι protein expression (Figure 2C). These results suggest that ζ-Stat is selective to PKC-ζ decreased expression and could be used to interrupt the PKC-ζ pathways.

### *In-silico* Results and Model Validation

#### Model Viability

RMSD and R_g_ plots of PKC-ι (Supplemental Figure 1) displayed asymptotic behavior beyond 7 ns. RMSD appeared to equilibrate near 2.3 Å, similar to the reported crystallographic resolution of 2 Å (24). Any additional fluctuation is likely from the disordered tail regions and missing residues 446–454 as evidenced from the PKC-ι RMSF plot (Supplemental Figure 2). From these analyses, the MD model is considered viable due to the ability of the model to accurately maintain the lowest energy structure under significant perturbation.

**Figure 3:**
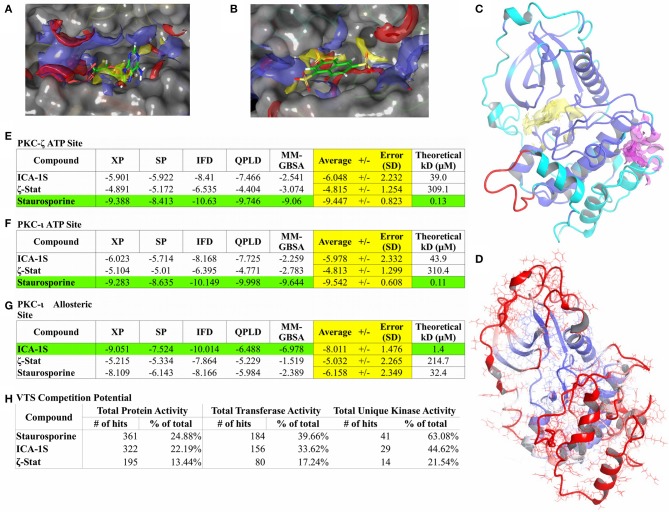
Computational modeling results. Binding site characterization for PKC-ι ATP site with co-crystallized ATP shown for reference **(A)** and the PKC-ι potential allosteric site with ζ-Stat docking result shown for reference **(B)**. SiteMap analysis for each site is also shown **(A,B)**. Yellow volume denotes hydrophobic regions, red volumes demarcate hydrogen bond donor sites, and blue volumes define hydrogen bond acceptor sites. The final homology model structure of PKC-ζ **(C)** generated is displayed with cartoon ribbons and colored by sequence identity to the PKC-ι reference structure. Blue are identical residues between both structures with cyan highlighting dissimilar amino acids. Red indicates sequence spans with no structural template. The ATP binding region is also delineated by a yellow domain and a magenta domain localizes the suspected allosteric site, both defined by SiteMap. Post-MD RMSD analysis **(D)** is illustrated with cartoon ribbons colored by RMSD per residue over the length of the simulation. Continuous color shift is used to show residue displacement from original structure with blue representing a minimum of 0 Å RMSD, white representing mid values of 4 Å RMSD, and red representing a maximum of 8 Å RMSD. Consensus docking scores **(E–G)** are measured in kcal/mol and represent estimated free energies of binding (ΔG°). Green highlighting features the best scoring molecule for a particular site and yellow highlight focuses the averaged consensus scoring for each compound. Theoretical K_D_ values are calculated from free energies of binding (ΔG°). VTS results are also listed **(H)**.

To date, no crystallographic structure exists of PKC-ζ. As such, a homology model was attempted using the Schrödinger program Prime (28–30) with the human PKC-ζ sequence (UniProt Q05513) and the crystallized PKC-ι structures as templates. PKC-ι was chosen as the primary template due to its highest sequence identity (49.0%) and homology (53.8%) given from a BLAST search (77) coupled with highest overall structural resolution. A pre-generated SWISS-MODEL (25–27) homology model was used as a control for structural reference. The Prime model (Figure 3C) was identical to the SWISS-MODEL variant, so only the Prime model was used for further analysis. RMSD and R_g_ plots of the PKC-ζ homology model (Supplemental Figure 3) do show asymptotic behavior after 1.2 ns, however the RMSD equilibration is averaged to 4.4 Å and is clearly outside any acceptable resolution. The R_g_ plot also shows a significant expansion indicative of unfolding with internal water infiltration. RMSF plots (Supplemental Figure 4) also show substantial backbone movement beyond 2 Å at regions of PKC-ζ residues differing from PKC-ι with notable disruption of predicted secondary structure. These factors conclude that the homology model does not represent a physical low energy structure and therefore cannot be used for further modeling. Despite this, the ATP binding region is largely stable with RMSF well within reasonable values (Figure 3D). This allows inclusion of the PKC-ζ homology model ATP binding site with docking studies to compare binding modes between PKC-ι and PKC-ζ ATP binding sites (Figure 3C—yellow region).

The Schrödinger program SiteMap was used for optimizing placement of grids used in the docking studies and to search for any other potential binding sites. A possible allosteric site for PKC-ι was identified by SiteMap near residues 397–400 in the activation segment of the PKC-ι c-lobe consisting of a pocket made by α-helices E and F and the interhelical loop between α-helices H and I (Figure 3C—magenta region). SiteMap also scores regions based on potential hydrogen bonding, hydrophobicity, and pocket volume. Scores of 0.8 or greater are considered the cutoff for distinguishing between drug-binding and non-drug-binding sites. The PKC-ι ATP site was scored at 1.004 and the potential allosteric site was scored at 0.779. The potential allosteric site score is notably within SiteMap calibration error and was still included in docking studies due to proximity of the pocket with the PKC-ι activation segment and for the possibility of induced fit effects opening the site.

#### Docking Results

Minimal direct binding data exists for PKC-ι and PKC-ζ so a consensus docking approach was utilized to gauge the optimal docking algorithm for the ATP and potential allosteric site. The approach detailed utilizes five different computational methods of discerning theoretical binding affinities: two unique scoring functions (SP and XP) for ridged docking methods, IFD to account for potential induced fit effects, QPLD can resolve polarization effects through QM/MM techniques, and MM/GBSA is superior in clarifying penalties for solvent interactions. Employing these functions when little empirical evidence exists to correlate results helps identify weaknesses of each technique for a particular model. Error can also be statistically minimized, yielding a more accurate summary of ligand binding dispositions and affinities.

Staurosporine was used as a docking control for the ATP site since binding data exists for both PKC-ι [261 nM K_i_ (78), values converted from IC_50_s] and PKC-ζ [131 nM K_i_ (79), values converted from IC_50_s]. ATP is not used as a docking control due to poor model forcefields and lack of direct binding data.

Docking results are summarized in Figures 3E–G. Docking scores for staurosporine controls are well within reasonable agreement with literature values. Docking scores and poses for each molecule are nearly identical for the ATP site of PKC-ι and PKC-ζ and both prefer staurosporine by a significant margin (Figures 3E,F, green highlight). XP scoring consistently yielded scores in closest agreement to literature values and highest Pearson correlation to overall averages. IFD and QPLD have poorer correlation and control accuracy, suggesting a less pronounced influence of charge factors and induced fit effects since including polarization and site flexibility does not increase docking accuracy. MM/GBSA scores exhibit similarly reduced correlation and high variance, entailing that solvent effects are also not likely a major factor for binding; this is understandable given the pocket depth. These analyses signify that the hydrophobic centers of the site (Figure 3A) are the dominant factors in ligand binding with the ATP site for both models. As opposed to the pan-kinase inhibitor, staurosporine, ICA-1S and ζ-Stat display negligible binding with the ATP site.

The potential allosteric site was also studied (Figures 3B,G), but only for PKC-ι since the corresponding PKC-ζ allosteric site model could not be validated. As such, no conclusions should be drawn concerning possible interactions of these compounds with any potential allosteric sites on PKC-ζ (only the ATP site achieved an apparent suitable stability for docking studies). Of the three molecules, the potential allosteric site appears to prefer only ICA-1S with a theoretical K_D_ of 1.4 μM. A recent study by Ratnayake et al. (19) measured myelin basic protein (MBP) phosphorylation by PKC-ι and PKC-ζ in the presence of ICA-1S, ICA-1T (the phosphorylated version of ICA-1S), and ζ-Stat. Docking figures match expected activity of PKC-ι for ζ-Stat having negligible inhibition. ICA-1S activity as a PKC-ι inhibitor gives some support to the existence of the potential allosteric site, since modeling suggests that ICA-1S does not interact significantly with the ATP site, but does display binding at concentrations similar to experimental values for inducing inhibition. Unfortunately, the data for PKC-ζ is less clear. All the modeling can show is if the two compounds in question can effectively bind to the ATP site of PKC-ζ. This may indirectly imply an allosteric site exists for PKC-ζ if inhibition is experimentally observed and the compound in question does not appear to have favorable docking to the ATP site. For ICA-1S, there is negligible affinity for the ATP site and experiment reflects a lack of inhibition. Modeling also suggests an allosteric mechanism may be present for ζ-Stat as binding is also negligible for the ATP site. Experimental inhibition should be observed for any compounds that compete for the ATP site since there is no significant difference between the ATP sites of PKC-ι and PKC-ζ.

#### Virtual Target Screening Results

VTS uses a large curated protein structure library to which molecules of interest are docked. Statistical calibrations and baselines are applied to average and relate docking scores with each individual and class of proteins. A kinase-enriched library (1,451 proteins, 464 transferases, and 65 unique kinases) was assigned for docking with the three compounds. A hit on a protein is classified as the potential (*p* < 0.05) of the molecule of interest to bind to the specified protein with a theoretical K_D_ of 10 μM or less. This analysis can infer the specificity of a molecule for a particular class of proteins. The VTS results for each compound are listed in Figure 3H.

The staurosporine control gave an expected baseline commensurate of a pan-kinase inhibitor. It displayed low to moderate total protein activity with moderately high interaction with general transferases and hit a majority of kinases, alluding a clear preference for kinases. ICA-1S demonstrated a low total protein activity with a slight but pronounced increase in affinity for transferases and kinases. This suggests a possibility of seeing some expected broad kinase interference for ICA-1S. ζ-Stat, however, portrays significant specificity in VTS. It has similar low hit percentages for all protein classes, implying little to no expected kinase activity.

### Inhibition of Cell Viability

**Figure 4:**
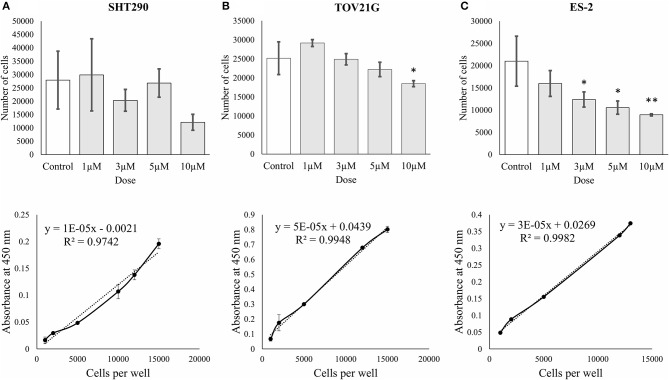
Cell proliferation and viability of SHT290, TOV21G, and ES-2 CCOC cell lines. **(A)** SHT290, **(B)** TOV21G, and **(C)** ES-2 cells were treated for 0, 24, 48 and 72 h with 1, 3, 5, and 10 μM ζ-Stat. WST-1 assays were run after 72 h of ζ-Stat treatment (Vehicle control *N* = 12, treatment *N* = 3). A standard curve for each cell line is also represented. Standard deviation is represented. A one-way ANOVA was tested for the WST-1 assays (*p < 0.05, **p < 0.01).

The effects of ζ-Stat on CCOC cellular viability was investigated via WST-1 methodologies. The results revealed that 10 μM ζ-Stat did not significantly effect SHT290 normal endometrial stromal cells, but did significantly decreased the viability by 37% in TOV21G cells (*p*-value 0.0436, F = 4.2461, *t* = −3.058) and by 57% in ES-2 cells (*p*-value 0.00363, F = 7.2918, *t* = −4.220) (Figures 4A–C). These results suggest that ζ-Stat decreases the viability of CCOC but has negligible effects on normal endometrial stromal cells.

### Analysis of the PKC-ζ/Ect2/Rac1/RhoA Pathway

**Figure 5:**
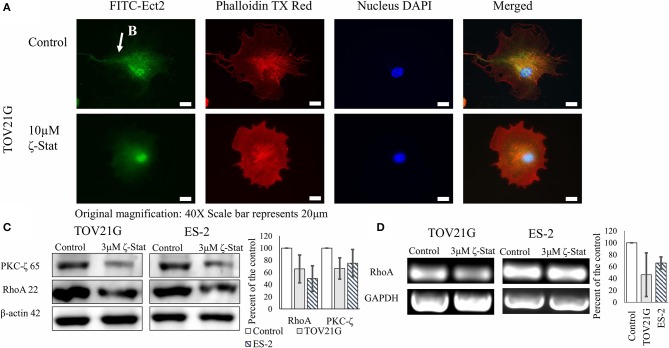
Effects of ζ-Stat on Ect2 localization and RhoA protein and genomic expression. **(A)** TOV21G cells were seeded on a four-chamber slide and treated for 72 h with 10μM ζ-Stat. The slide was fixed with formaldehyde and immunostained with anti-Ect2, phalldoidin dye, and DAPI. Original magnification is 40X and scale bar is 20μm. **(B)** Ect2 is visualized in the filamentous extension of the TOV21G cell. **(C)** A Western Blot for TOV21G and ES-2 cells treated for 72 h with vehicle control (DMSO) and 3μM ζ-Stat is represented with an immunoblot that was probed for PKC-ζ and RhoA (*N* = 3). **(D)** TOV21G and ES-2 cells were treated for 72 h with 3 μM ζ-Stat and harvested with RNAbee for mRNA semi-quantification. Endpoint PCR was run with RhoA and GAPDH primers (mRNA control) (*N* = 3).

To determine the downstream effects of ζ-Stat on invasion, immunofluorescence, Western Blots and semi-quantitative endpoint PCR techniques were utilized. Vehicle control and ζ-Stat treated TOV21G cells were probed with anti-Ect2 and imaged. The results showed that Ect2 was present in the filamentous extensions in control cell. After treatment, the polarity of the filamentous extensions decreased and Ect2 was found to be more abundant around the nucleus (Figures 5A,B). Western results demonstrated that 3 μM of ζ-Stat decreased RhoA protein expression as well as mRNA expression (Figures 5C,D). These results suggest that the decrease in PKC-ζ protein expression reduces the expression of RhoA at the genomic level.

**Figure 6:**
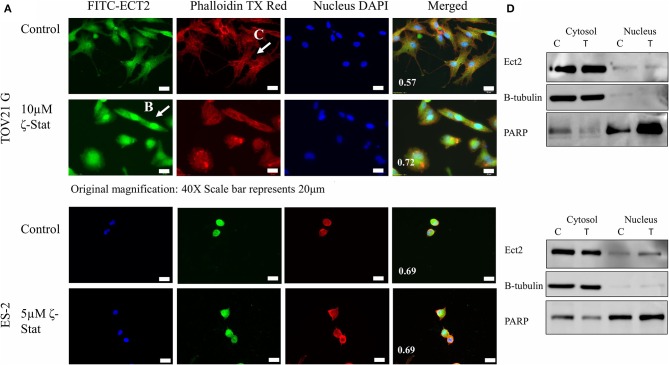
Localization of Ect2. **(A)** TOV21G and ES-2 cells were probed with anti-Ect2, phalloidin dye and DAPI. The Pearson's correlation coefficient is represented in white at the bottom left corner of the merged images. Original magnification is 40 × and scale bar is 20μm. **(B)** A visualization of the organized filamentous actin in TOV21G cells treated with vehicle control (DMSO). **(C)** A visualization of Ect2 localization in TOV21G cells treated with 10μM ζ-Stat for 72 h. **(D)** Nuclear and cytosolic fractionation of TOV21G and ES-2 cells were treated for 72 h with 10μM of ζ-Stat, Western blotted and immunoblotted with Ect2. Control antibodies used were β-tubulin (cytosol) and PARP (nucleus).

Furthermore, Ect2 localization was observed by immunofluorescence with and without treatment. The PCC showed that the control had a lower amount of Ect2 nuclear localization (0.57) in comparison to the treated (0.72) TOV21G cells (Figure 6A). In contrast, ES-2 cells had little effect as both the control and the treated cells had a PCC value of 0.69 (Figure 6B). In addition, the filamentous actin (F-actin) organization was investigated via phalloidin stain. In Figure 6C, the F-actin in the control for TOV21G showed filamentous extensions, whereas in the treated cells, the F-actin seemed to aggregate within the cell, rounding the edges. Although a nuclear fractionation could not confirm the translocation of Ect2 into the nucleus is TOV21G cells (Figure 6D), it did show that this translocation did occur in ES-2 cells. An explanation for this may be that the ζ-Stat is disrupting the cell structure causing the cells to become more globular and thereby making the cytosol surround the nucleus in a more significant manner.

**Figure 7:**
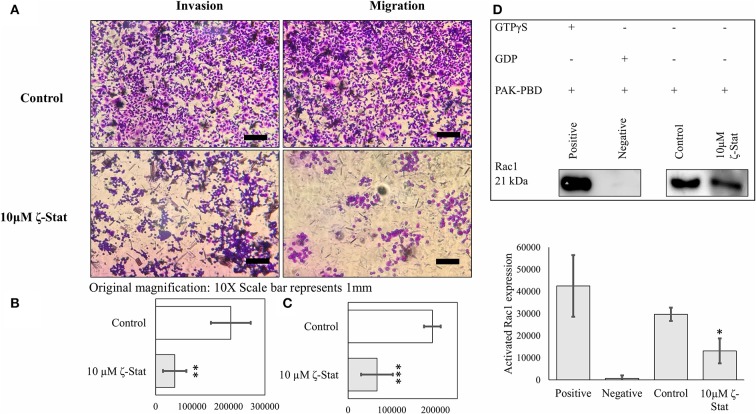
PKC-ζ regulates invasion and migration of TOV21G ovarian cancer. **(A)** TOV21G cells were grown, serum starved for 48 h, and placed in the upper chamber of transwell plate coated with 0.1x BME and serum (10%) containing media was placed in the lower chamber as a chemo attractant. Following treatment with 10μM ζ-stat for 24 h, cells that invaded through BME and migrated into the lower chamber were stained with crystal violet and observed under microscope. Original magnification is 10x and scale bar represents 1 mm. **(B)** Invasion (*N* = 4) and **(C)** migration (*N* = 4) fixed and stained cells on the lower chamber were quantified using ImageJ FIJI software, average and plotted. Standard deviation error bars are represented. (**p < 0.01, ***p < 0.001). **(D)** Activated Rac1 (GTP bound) was pulled down using GST tagged PAK-PBD and analyzed by Western Blot. Densitometry of activated Rac1 bands were plotted (*N* = 4). Standard deviation error bars are represented.

Moreover, TOV21G cells were treated with 10 μM ζ-Stat and seeded into 96 welled transwell plates. After 24 h of treatment, the cells were fixed and stained to determine the effects ζ-Stat on invasion and migration. Our data showed that ζ-Stat drastically decreased invasion and migration when compared to the control (Figure 7A). After the images were quantified, the data revealed that the decrease in invasion and migration was statistically significant (Figures 7B,C; invasion *p*-value 0.002826, *t* = 4.859; migration *p*-value <0.001, *t* = 6.1887). To further illustrate this point, Rac1 activation was investigated utilizing a GST pull down method. The negative and positive control display how well the assay data fits the intended model (*p*-value <0.001, *t* = 7.675). Compared to the sample control, the amount of activated Rac1 pulled down from treated samples was only 37% (*p*-value 0.044, *t* = −3.044; Figure 7D). These data reinforce the theory that ζ-Stat decreases the invasion and migration of CCOC through a decreased activation of Rac1.

### Analysis of ζ-Stat in TOV21G Tumor Xenografts

**Figure 8:**
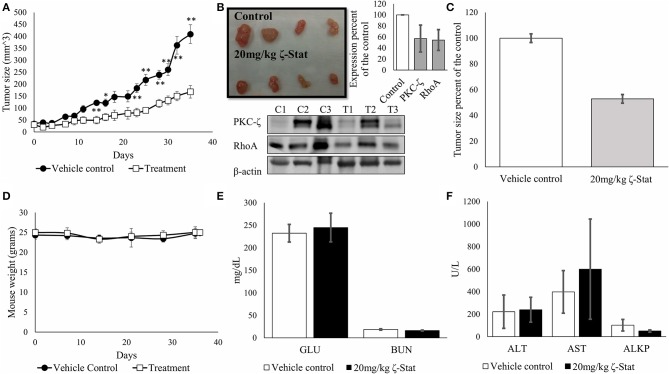
Effects of ζ-Stat on *in-vivo* xenografts with athymic nude mouse models. **(A)** Six vehicle control mice and six treatment mice were injected with 1 × 10^6^ cells/per mouse flank in 0.2 mL of media of TOV21G CCOC cells. Vehicle control mice tumors were treated with 1 × DPBS and treatment mice were treated with 20 mg/kg of ζ-Stat. Tumors were measured every other day and plotted. An unpaired student T test was performed, and standard deviation error bars are presented (*p < 0.05, **p < 0.01). **(B)** Tumors were harvested after 35 days of treatment and a picture was taken. Tumors were fractionated and protein lysates were run via Western blot. Analysis of the protein expression of PKC-ζ and RhoA were investigated. B-actin was used as a control. Densitometry of the results are represented in bar graph as expression percent of the control with standard deviation error bars. **(C)** Endpoint treated tumor volumes were graphed as a percent of the control **(D)** Mouse weight was recorded once per week. An unpaired student *T* test was performed, and standard deviation error bars are presented (no significant difference observed). **(E,F)** Endpoint blood serum was taken and screened for glucose (GLU), blood urea nitrogen (BUN), alanine aminotransferase (ALT), aspartate aminotransferase (AST) and alkaline phosphatase levels for toxicity. A unpaired student T test was performed, and standard deviation error bars are presented (no significant difference observed).

To determine the effects of ζ-Stat *in-vivo*, we injected athymic nude female mice with TOV21G cells and sequentially treated mice for 35 days. At the endpoint of the experiment, the tumors were harvested, and the blood serum was screened for enzymes associated with kidney and liver failure, as well as glucose levels for screening diabetes. Our data exhibited statistically significant changes in tumor volume between vehicle control and treated mice (Figure 8A) starting on day 14 (*p*-value 0.006343, *t* = 3.4389) up until day 35 (*p*-value 0.001136, *t* = 4.4827). Results also demonstrated that ζ-Stat decreased tumor growth by more than 50% by the endpoint of the experiment (Figures 8B,C) and decreased PKC-ζ and RhoA expression in tumors by more than 40%. The treatments did not lower the mouse population's body weight (Figure 8D) and did not have a significant effect on the enzyme panel (Figures 8E,F). These preliminary results suggest that ζ-Stat can be used for the treatment of CCOC and does not cause short-term toxicity.

### ζ-Stat Interrupts the PKC-ζ/Ect2 via PKC-ζ Protein Decrease

**Figure 9:**
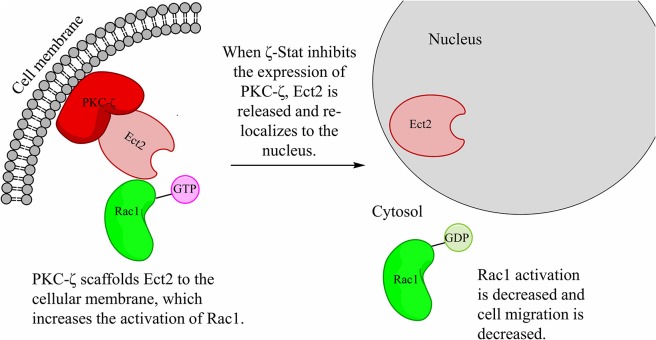
ζ-Stat decreases the amount of PKC-ζ, therefore decreasing the scaffold of Ect2 in the cytoplasm. The hypothesized pathway illustrates the scaffolding of Ect2 via PKC-ζ in the cytoplasm. Treatment with ζ-Stat decreases PKC-ζ expression thereby there is less PKC-ζ to bind Ect2, allowing Ect2 to re-localize to the nucleus. This decreases Ect2 cellular access to Rac1, reducing Rac1 activation and cell migration.

Our predicted pathway models that PKC-ζ scaffolds Ect2 to the cellular membrane (Figure 9). This mis-localization of Ect2 permits the wild-type Ect2 more access to Rac1 and therefore increases its activation. Upon increased Rac1 activation, CCOC invasion is increased. PKC-ζ protein level decrease via ζ-Stat, releases Ect2 from the membrane scaffold, and re-localizes Ect2 to the nucleus, limiting its access to cytosolic Rac1 and decreasing Rac1 activation.

## Discussion

In this study, we discovered that the small molecule inhibitor, ζ-Stat, is a prospective drug candidate to investigate as a novel potential treatment for CCOC. We also investigated the PKC-ζ/Ect2/Rac1 activation pathway and found that ζ-Stat decreases the invasive behavior of CCOC by decreasing cytosolic Ect2 and Rac1 activation.

### Targeting aPKCs in CCOC

To understand the therapeutic potential of a protein target, there must be an appreciation of the underlying genetic abnormalities specific to the cancer type. The TP53 gene (tumor suppressor p53) is the most commonly mutated gene in all ovarian cancers and is especially a prognostic marker of HGSOC (80). In contrast, CCOC typically has a wild type TP53 and mutations in the tumor suppressor ARID1A (81). Although PRKCZ and PRKCI are not in the top mutated genes in CCOC, PIK3CA is mutated (~30%) and is located on the third chromosome's long arm. Interestingly, PIK3CA (3q26.32), ECT2 (3q26.31), and PRKCI (3q26.2) are all located on the long arm of chromosome three and in proximity. The ARID1A (1p36.11) is also located up stream of PRKCZ (1p36.33) on the short arm of the first chromosome. It has been noted that mutations and deficiencies in ARID1A have been shown to sensitize cancers to PARP and PI3K inhibitors (82–84).

Previous literature has suggested that the atypical PKCs and their pathways should be the focus of targeted treatment (4). One explanation for this is that the atypical PKC isoforms have been linked to signaling pathways needed for cancer survival and growth. A study performed by Yao et al. illustrated the dramatic changes to prostate cells malignancy upon PKC-ζ silencing (85). In our study, when CCOC (TOV21G and ES-2) cells grew rapidly and were cell cycle arrested (serum starved), the aPKCs were present in both conditions. However, the expressions of PKC-ζ and PKC-ι were found to be very low in the normal endometrial stromal cells. These findings may indicate that CCOC cells have a reliance on aPKC overexpression for cell viability. An interesting complication in other types of CCOC is that ζ-Stat does not specifically limit expression of PKC-ζ but also effects downstream targets in the PKC-ζ/Ect2/RhoA pathway. As shown in Figure 2, ζ-Stat had a negligible effect on PKC-ζ expression in ES-2 cells, however in Figure 5, there was a decrease in RhoA protein and mRNA expression. This leads to the conclusion that ζ-Stat has generalized effect on the pathway dependent on cell type. Furthermore, these data support that PKC-ζ/Ect2/RhoA pathway contains relevant targets for CCOC due to the lack of overexpression in normal tissue and the overexpression in cancerous cell lines.

Equally important, a previous study showed that the knock down of PKC-ζ using siRNA decreased the expression of RhoA and Rac1 (86). Furthermore, this study illustrated that the knock down of PKC-ζ decreased the invasive behavior of breast cancer cells by more than 40%. Our data supports previous data and reiterates the therapeutic potential for targeting PKC-ζ in CCOC.

### aPKCs Are Involved in the Localization of Ect2

The mis-localization and overexpression of Ect2 has been linked to aPKCs and Ect2 dependent malignant transformation (87). Cytoplasmic Ect2 has more access to Rho GTPases and increases the protein family's activation. Liu et al. identified Ect2 as an activator of the Par6/Par3/aPKC polarity complex and further showed that Ect2 stimulated PKC-ζ activity (88). Moreover, the oncogenic activity of Ect2 was shown to be regulated by aPKC via the phosphorylation of the Thr 328 site and a mutation in this site (T328A) rendered the Ect2 unable to interact with Par6/aPKC complex or activate Rac1 (89). Upon treatment with ζ-Stat, the decrease of PKC-ζ disturbs the localization of Ect2 in TOV21G cells. This specific effect is not seen in ES-2 as correlations are equal before and after ζ-Stat treatment. Therefore, it does not seem that PKC-ζ is the specific target but more of the generalized PKC-ζ/Ect2/RhoA pathway.

### ζ-Stat Has Therapeutic Potential in CCOC

A common treatment regime for ovarian cancer patients typically involves chemotherapy (paxitaxol), PARP (poly ADP ribose polymerase) inhibitors and bevacizumab (targets angiogenesis) (11). However, concerns for current treatments still involve toxicity, drug-resistance, reoccurrence, and the side effects to the patients (90). Our data showed that mice treated with ζ-Stat did not have significant side effects when compared to mice treated with the vehicle control. In particular, the mice did not have significant fluctuations in body weight, or differences in the enzyme panel which screened for liver and kidney damage. Further, the viability of normal endometrial stromal cells did not change significantly upon treatment of 10 μM ζ-Stat. All these data support the prospect that this compound may be a less toxic alternative maintenance drug for CCOC.

The presence of PKC-ζ and PKC-ι in proliferation, invasion and migration make this protein a unique target for therapies. In the previous literature, ζ-Stat was shown to decrease the invasion and migration of melanoma cells and increase apoptosis (19). It has also been suggested the ζ-Stat is selective to PKC-ζ in melanoma and colorectal cell lines (16, 19, 91). In support of the previous data, our data showed that ζ-Stat had a dramatic effect on CCOC invasion and migration.

However, new evidence supports the theory that ζ-Stat does not inhibit kinase activity of PKC-ζ through the ATP binding region. Our computational and *in-vitro* data advocates that ζ-Stat decreases the expression of signaling proteins (PKC-ζ and RhoA). This phenotypic effect consistently modifies kinase interaction networks which results in decreases in cellular viability, invasion and tumor growth (16, 19). The effects that ζ-Stat elicited was decreased PKC-ζ protein, re-localized Ect2 to the nucleus and decreased the presence of activated Rac1. ζ-Stat specifically decreased the protein expression of PKC-ζ in comparison to PKC-ι, however this mechanism is still unknown. The data suggests that ζ-Stat may be generating an epigenetic effect, which in turn regulates the expression of these proteins. It has been suggested that using aPKC inhibitors can epigenetically regulate the expression of aPKCs through transcription factors, such as FOX01 (92).

Additionally, according to the computational data, ICA-1S exhibited poor binding with the PKC-ι and PKC-ζ ATP site and had a predicted moderate affinity with a possible PKC-ι allosteric site. This fits well with previously performed MBP phosphorylation experiments (19). Some expected kinase activity with mid-range binding promiscuity is anticipated for this molecule. However, this conclusion is contradicted by some evidence from the experiment, therefore it could simply be that the generated PKC-ζ model is insufficient for correlating physical data. ζ-Stat exhibits extremely poor binding with the ATP site and PKC-ι allosteric site. With no expected kinase activity and low binding promiscuity, any inhibition seen using ζ-Stat is likely specific for unique binding pockets.

To further the exploration of ζ-Stat being a potential therapeutic for CCOC, a mechanistic understanding of the direct protein binding is required for PKC-ζ and PKC-ι.

## Data Availability Statement

The datasets used and/or analyzed during the current study are available from the corresponding authors on reasonable request. The dataset generated and/or analyzed during the current study are available from COSMIC repository, https://cancer.sanger.ac.uk/cosmic.

## Ethics Statement

The animal study was reviewed and approved by Adrienne Booker Institutional Animal Care and Use Committee.

## Author Contributions

TS contributions include cell culture, analysis of somatic gene mutations, cell viability assays, cell lysate collection, Western Blot analysis, semi-quantitative endpoint PCR, Rac1 activation assay, fluorescent microscopy, *in-vivo* experiments, and the statistical analysis of Western Blots, cell viability, particle counts for invasion and migration, tumor volume, mouse body weights, and enzyme panel. In addition, TS contributed to writing and experimental design. RM contributed by providing molecular dynamics simulations, homology modeling, molecular docking, virtual target screening, site screening, computational and statistical analysis, and writing support. RP contributed by assisting *in-vivo* experimentation. SI contributed by performing the invasion and migration assay. RB contributed by performing Western blots. MA-D contributions to the paper include writing-review, editing, resources, supervision, and funding acquisition. The authors read and approved the final manuscript.

### Conflict of Interest

The authors declare that the research was conducted in the absence of any commercial or financial relationships that could be construed as a potential conflict of interest.
