# Supplementary material for: The Atypical Protein Kinase C Small Molecule Inhibitor ζ-Stat, and Its Effects on Invasion Through Decreases in PKC-ζ Protein Expression
Source: Front Oncol. 2020 Feb 27;10:209. doi: 10.3389/fonc.2020.00209 (PMC7056911; doi:10.3389/fonc.2020.00209)
Supplement: Supplementary file 1 [file Presentation_1.PPTX]

## Slide 1
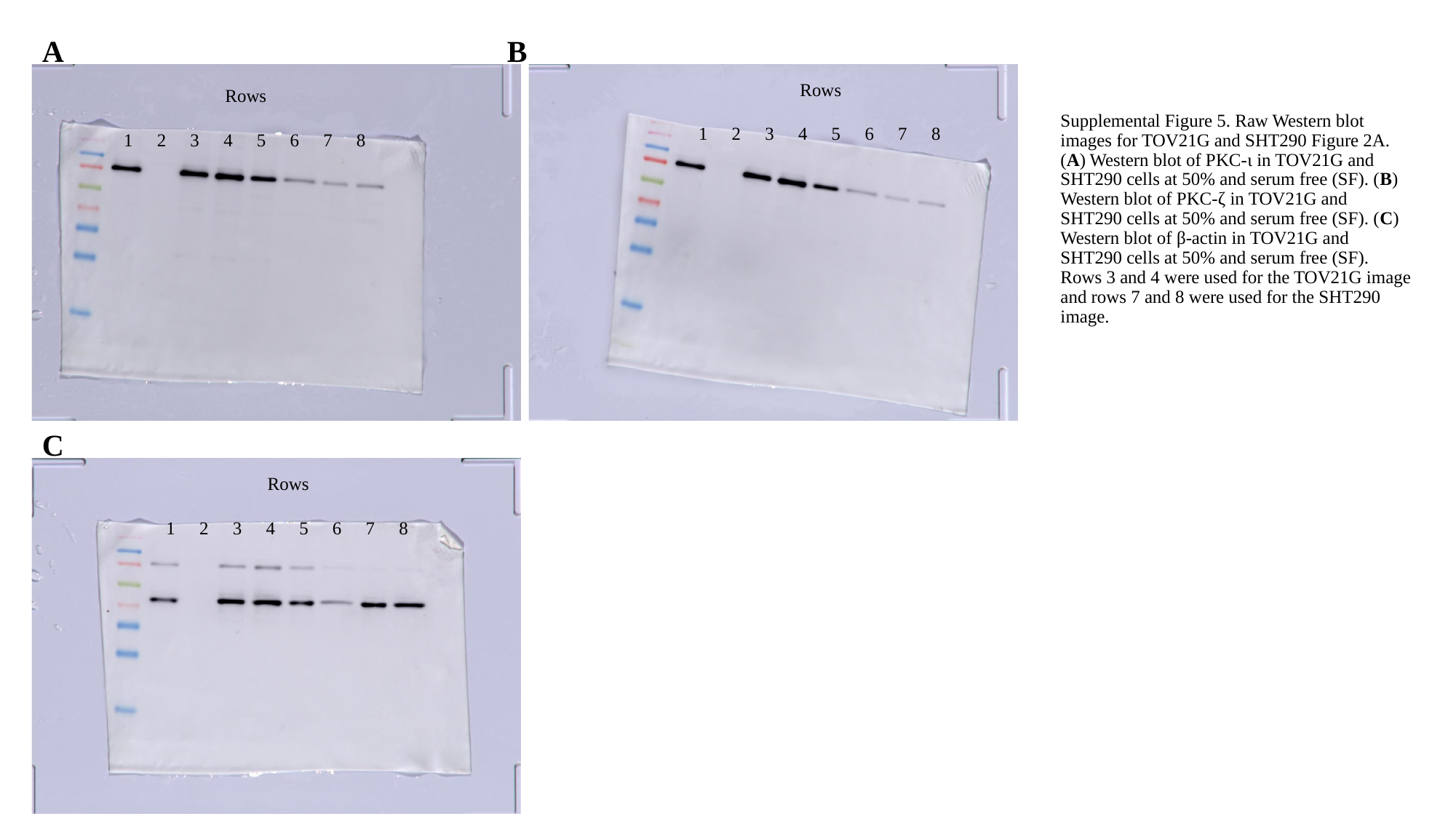

A
B
| Rows | | | | | | | |
| --- | --- | --- | --- | --- | --- | --- | --- |
| 1 | 2 | 3 | 4 | 5 | 6 | 7 | 8 |
| Rows | | | | | | | |
| --- | --- | --- | --- | --- | --- | --- | --- |
| 1 | 2 | 3 | 4 | 5 | 6 | 7 | 8 |
Supplemental Figure 5. Raw Western blot images for TOV21G and SHT290 Figure 2A. (A) Western blot of PKC-ι in TOV21G and SHT290 cells at 50% and serum free (SF). (B) Western blot of PKC-ζ in TOV21G and SHT290 cells at 50% and serum free (SF). (C) Western blot of β-actin in TOV21G and SHT290 cells at 50% and serum free (SF). Rows 3 and 4 were used for the TOV21G image and rows 7 and 8 were used for the SHT290 image.
C
| Rows | | | | | | | |
| --- | --- | --- | --- | --- | --- | --- | --- |
| 1 | 2 | 3 | 4 | 5 | 6 | 7 | 8 |

## Slide 2
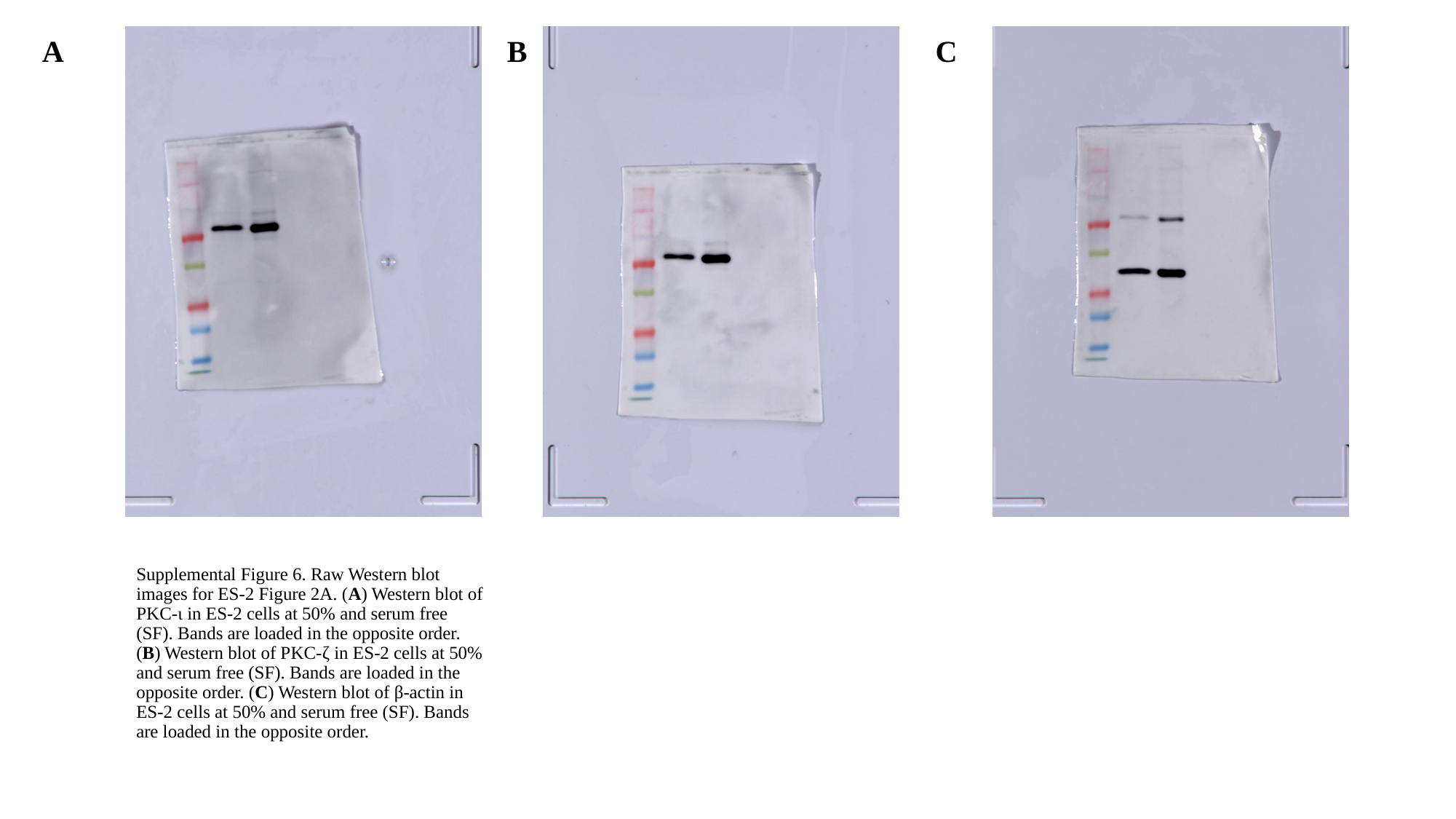

A
B
C
Supplemental Figure 6. Raw Western blot images for ES-2 Figure 2A. (A) Western blot of PKC-ι in ES-2 cells at 50% and serum free (SF). Bands are loaded in the opposite order. (B) Western blot of PKC-ζ in ES-2 cells at 50% and serum free (SF). Bands are loaded in the opposite order. (C) Western blot of β-actin in ES-2 cells at 50% and serum free (SF). Bands are loaded in the opposite order.

## Slide 3
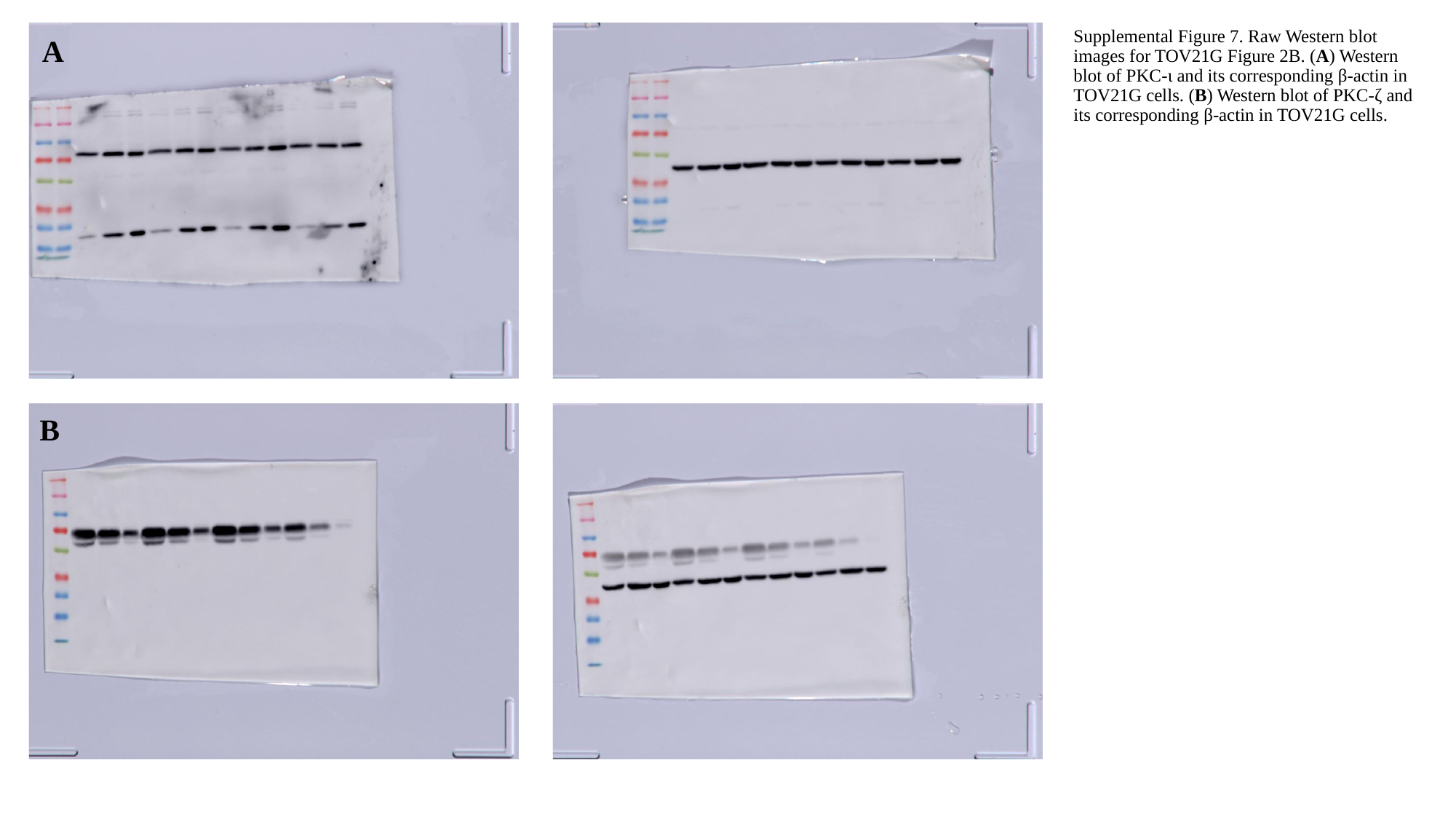

Supplemental Figure 7. Raw Western blot images for TOV21G Figure 2B. (A) Western blot of PKC-ι and its corresponding β-actin in TOV21G cells. (B) Western blot of PKC-ζ and its corresponding β-actin in TOV21G cells.
A
B

## Slide 4
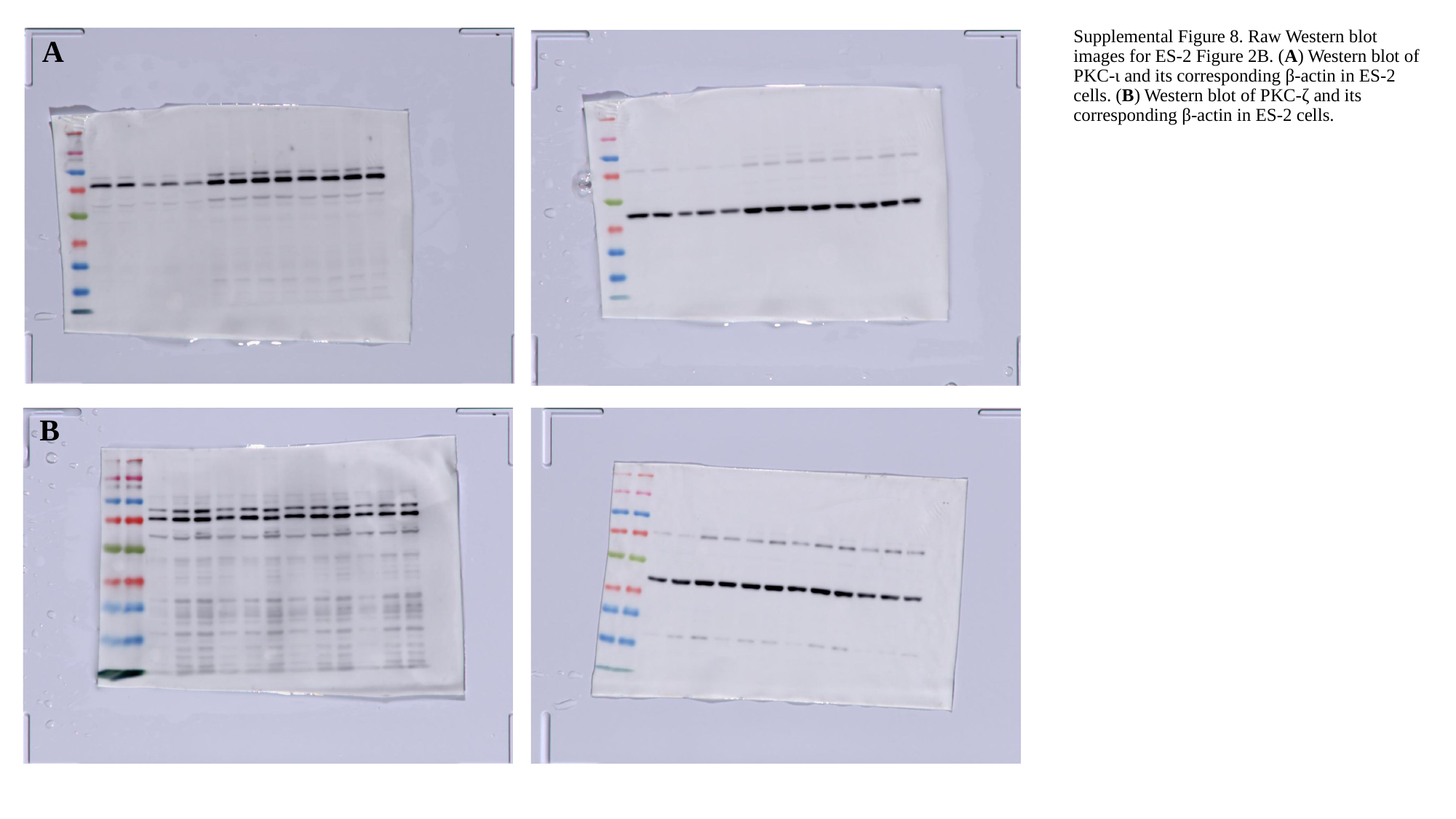

Supplemental Figure 8. Raw Western blot images for ES-2 Figure 2B. (A) Western blot of PKC-ι and its corresponding β-actin in ES-2 cells. (B) Western blot of PKC-ζ and its corresponding β-actin in ES-2 cells.
A
B

## Slide 5
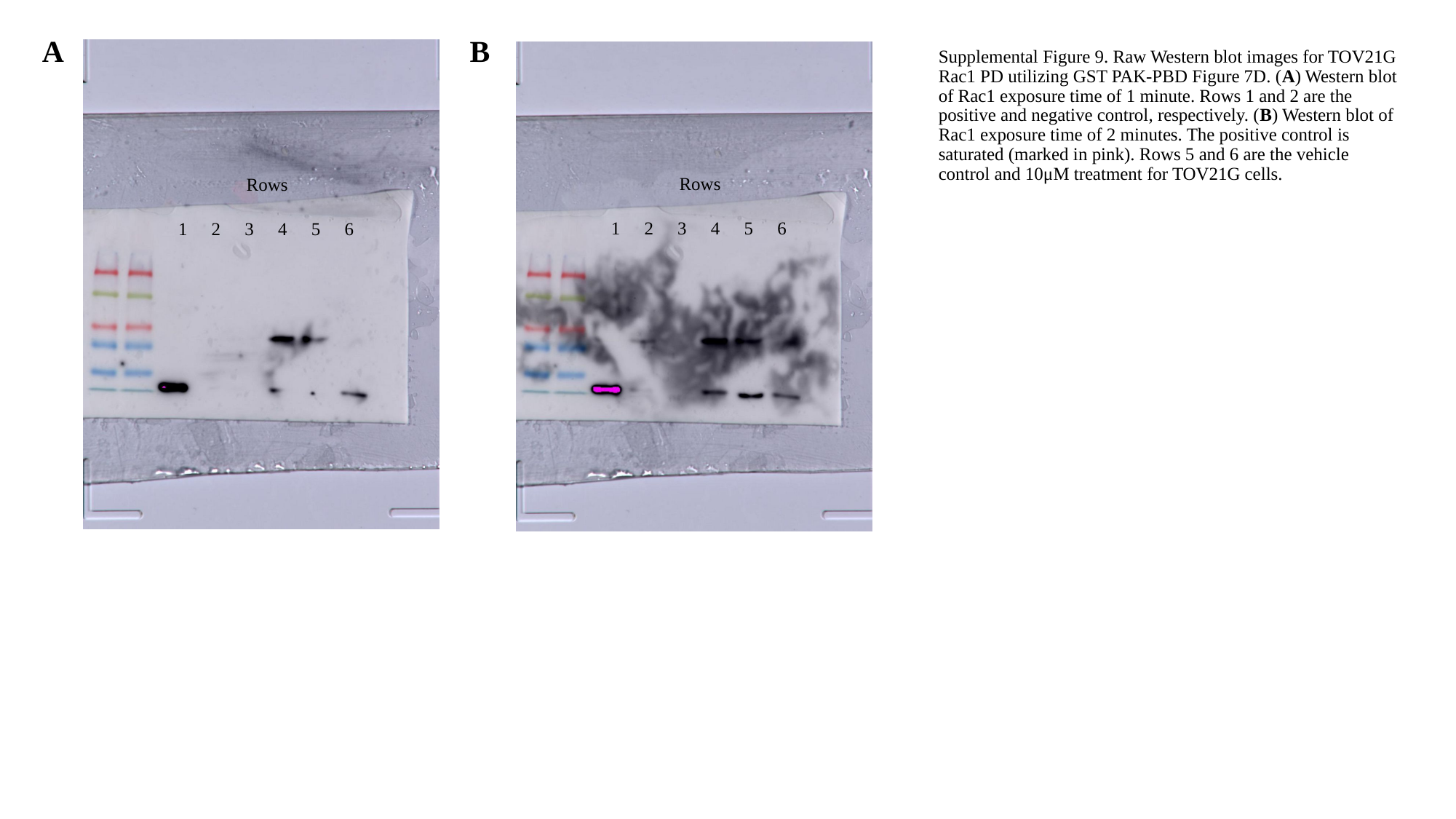

A
B
Supplemental Figure 9. Raw Western blot images for TOV21G Rac1 PD utilizing GST PAK-PBD Figure 7D. (A) Western blot of Rac1 exposure time of 1 minute. Rows 1 and 2 are the positive and negative control, respectively. (B) Western blot of Rac1 exposure time of 2 minutes. The positive control is saturated (marked in pink). Rows 5 and 6 are the vehicle control and 10μM treatment for TOV21G cells.
| Rows | | | | | |
| --- | --- | --- | --- | --- | --- |
| 1 | 2 | 3 | 4 | 5 | 6 |
| Rows | | | | | |
| --- | --- | --- | --- | --- | --- |
| 1 | 2 | 3 | 4 | 5 | 6 |

## Slide 6
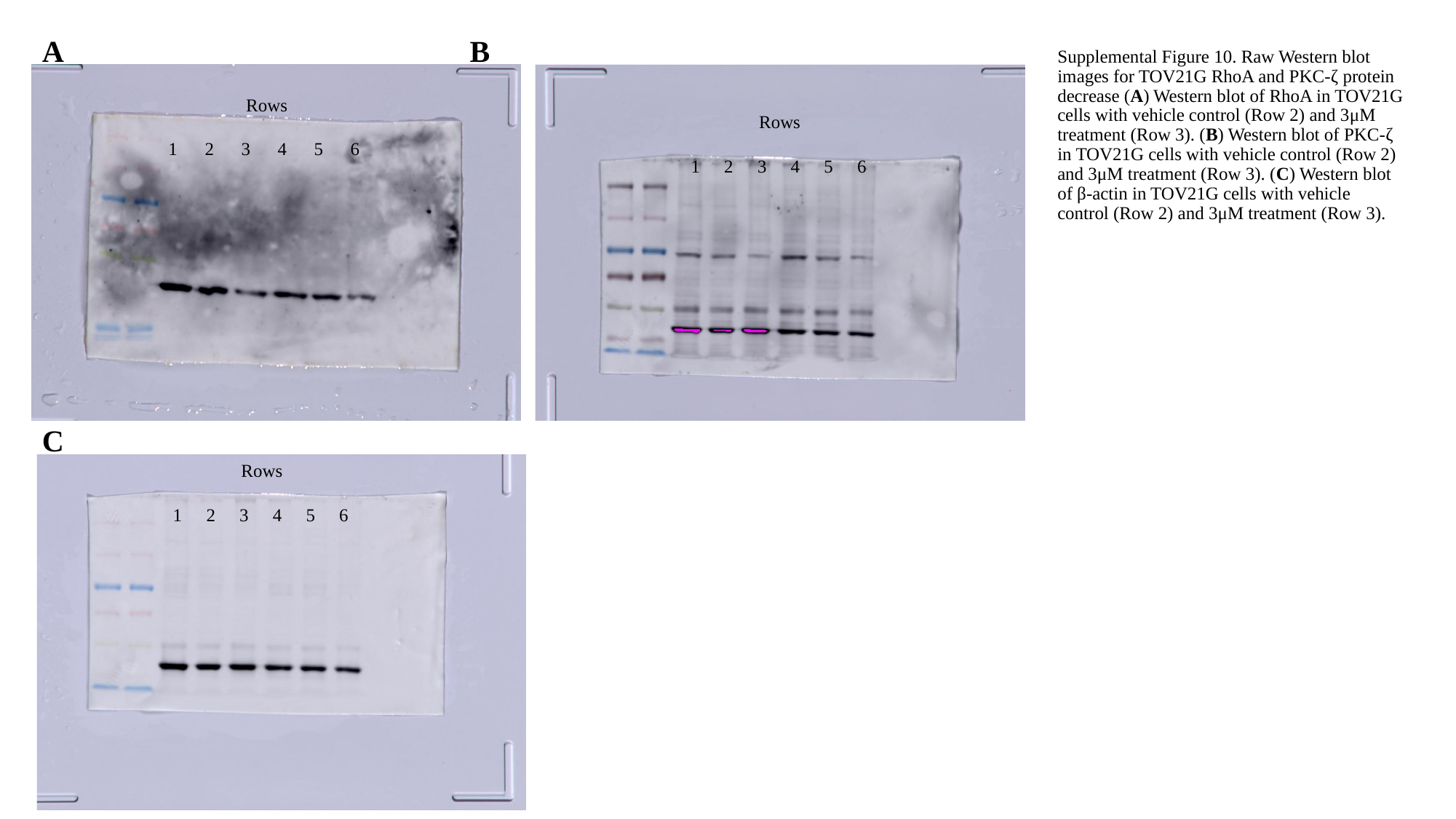

A
B
Supplemental Figure 10. Raw Western blot images for TOV21G RhoA and PKC-ζ protein decrease (A) Western blot of RhoA in TOV21G cells with vehicle control (Row 2) and 3μM treatment (Row 3). (B) Western blot of PKC-ζ in TOV21G cells with vehicle control (Row 2) and 3μM treatment (Row 3). (C) Western blot of β-actin in TOV21G cells with vehicle control (Row 2) and 3μM treatment (Row 3).
| Rows | | | | | |
| --- | --- | --- | --- | --- | --- |
| 1 | 2 | 3 | 4 | 5 | 6 |
| Rows | | | | | |
| --- | --- | --- | --- | --- | --- |
| 1 | 2 | 3 | 4 | 5 | 6 |
C
| Rows | | | | | |
| --- | --- | --- | --- | --- | --- |
| 1 | 2 | 3 | 4 | 5 | 6 |
