# Supplementary material for: The Atypical Protein Kinase C Small Molecule Inhibitor ζ-Stat, and Its Effects on Invasion Through Decreases in PKC-ζ Protein Expression
Source: Front Oncol. 2020 Feb 27;10:209. doi: 10.3389/fonc.2020.00209 (PMC7056911; doi:10.3389/fonc.2020.00209)
Supplement: Supplementary file 2 [file Data_Sheet_1.PDF]

Molecular Dynamics results

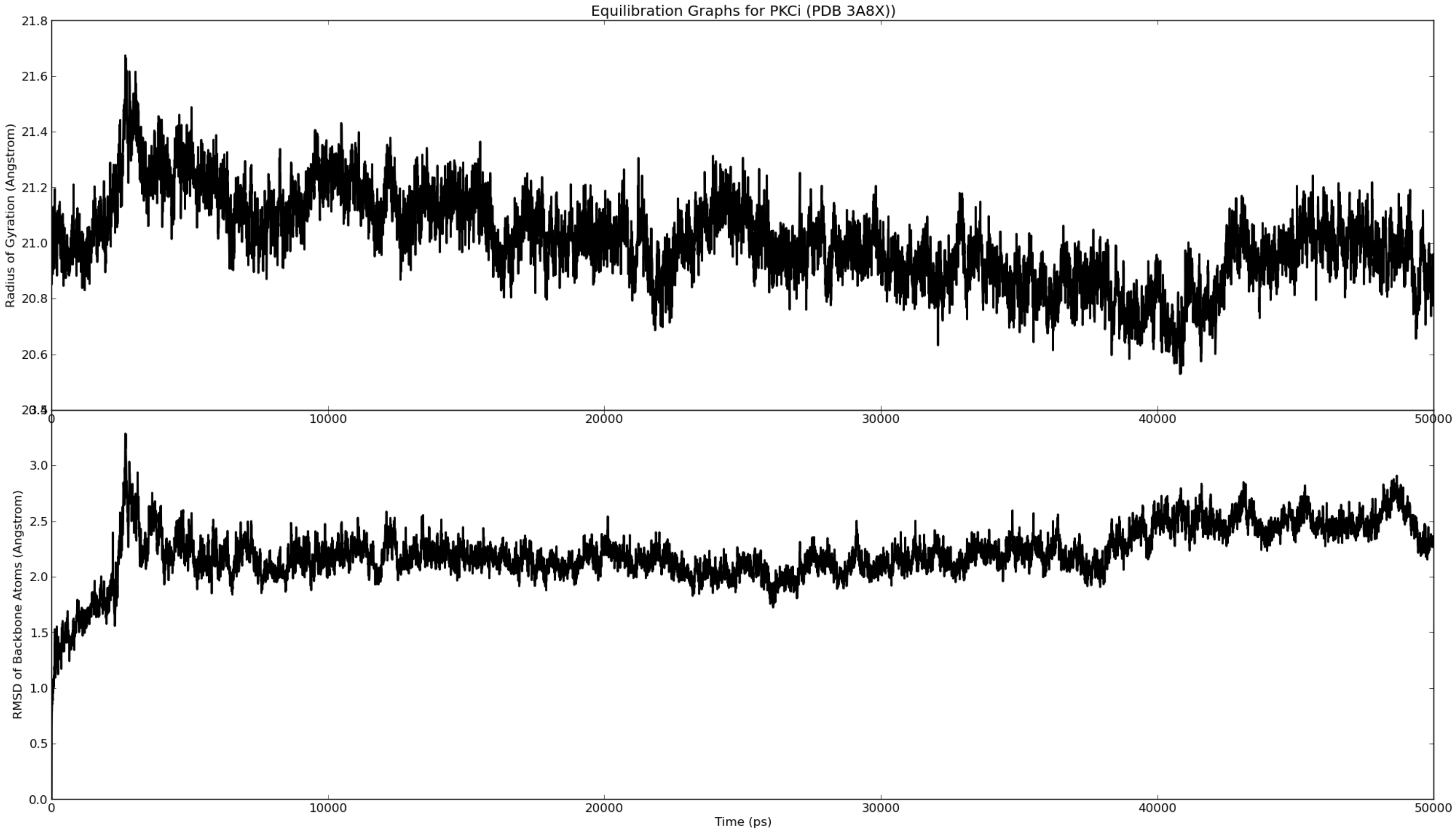

Supplemental Figure 1.) RMSD (Bottom) of backbone atoms and Radius of Gyration (R_g, Top) of all atoms graphs for PKCι Molecular Dynamics simulation. Equilibration appears to occur after 7 ns with an average RMSD of 2.3 Å.

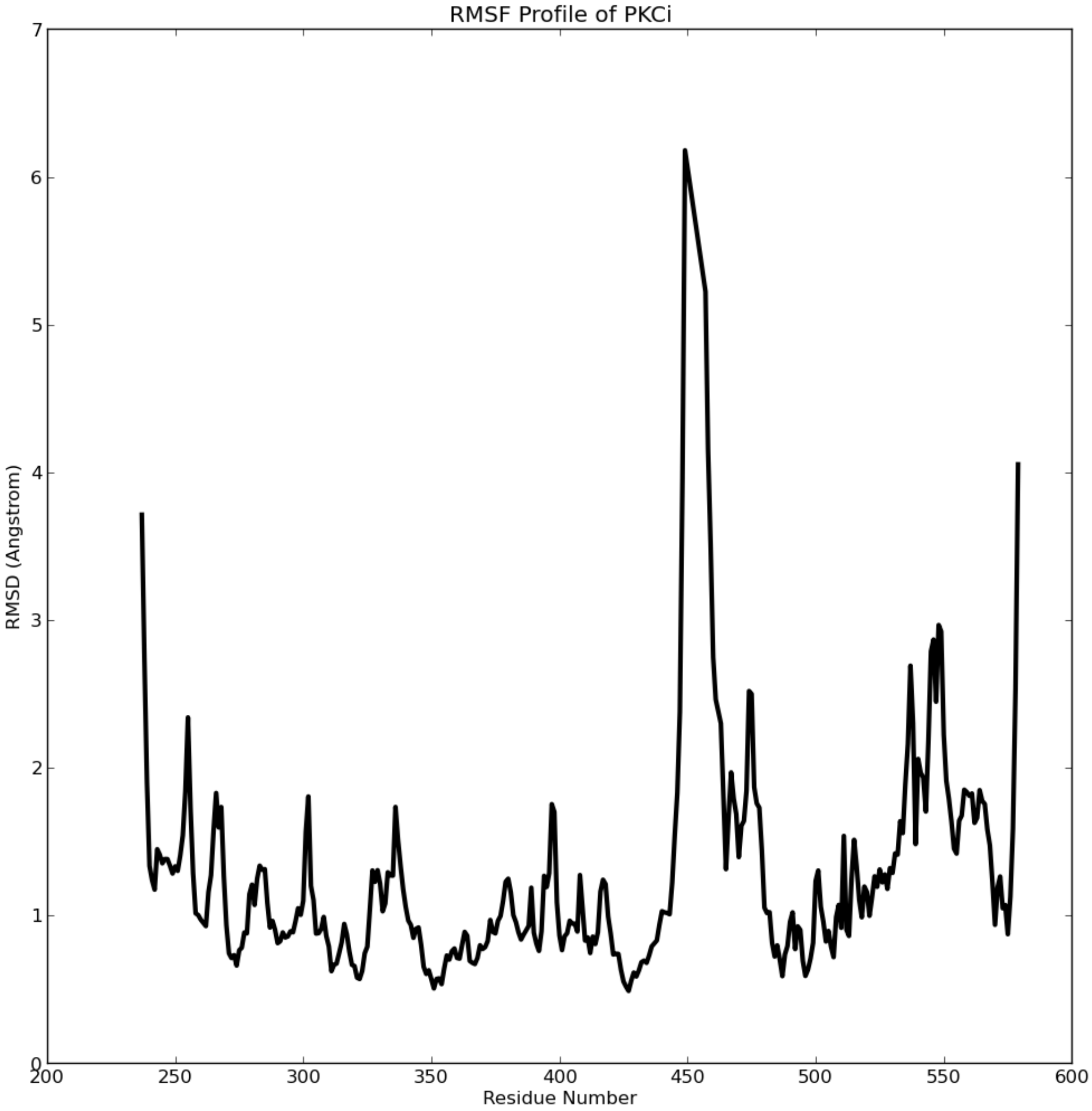

Supplemental Figure 2.) RMSF (Root Mean Square Fluctuation) plot for PKCι Molecular Dynamics simulation. Graph shows RMSD per residue of α-carbon over length of simulation. Most displacements are under 2 Å aside from tail regions and chain discontinuities from missing residues 446 – 454.

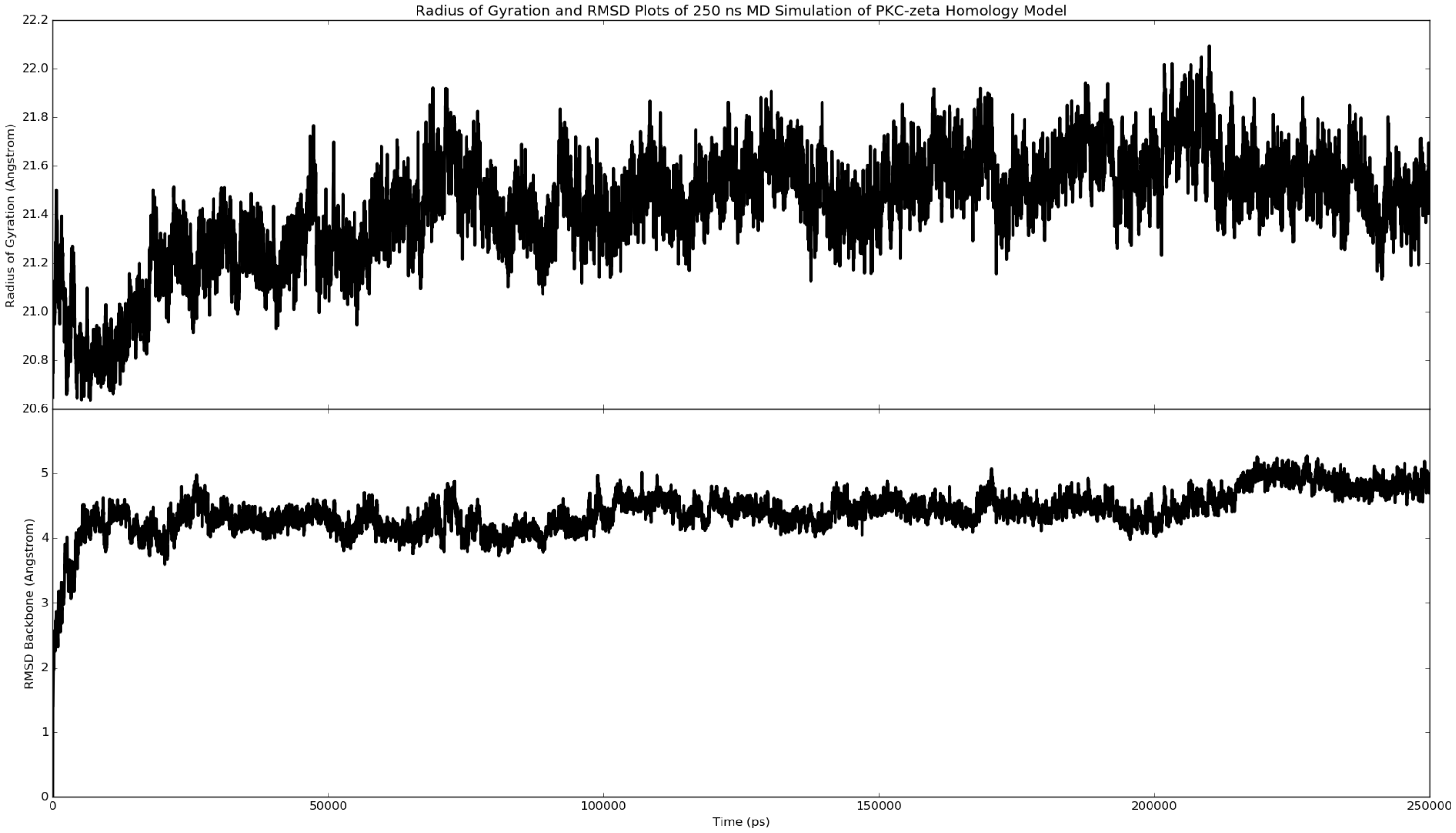

Supplemental Figure 3.) RMSD (Bottom) of backbone atoms and Radius of Gyration (R_g, Top) of all atoms graphs for PKCζ Molecular Dynamics simulation. Some asymptotic behavior does appear to occur, however, an average RMSD of 4.5 Å and increasing R_g implies unfolding and significant deviation from any structural energetic minimum.

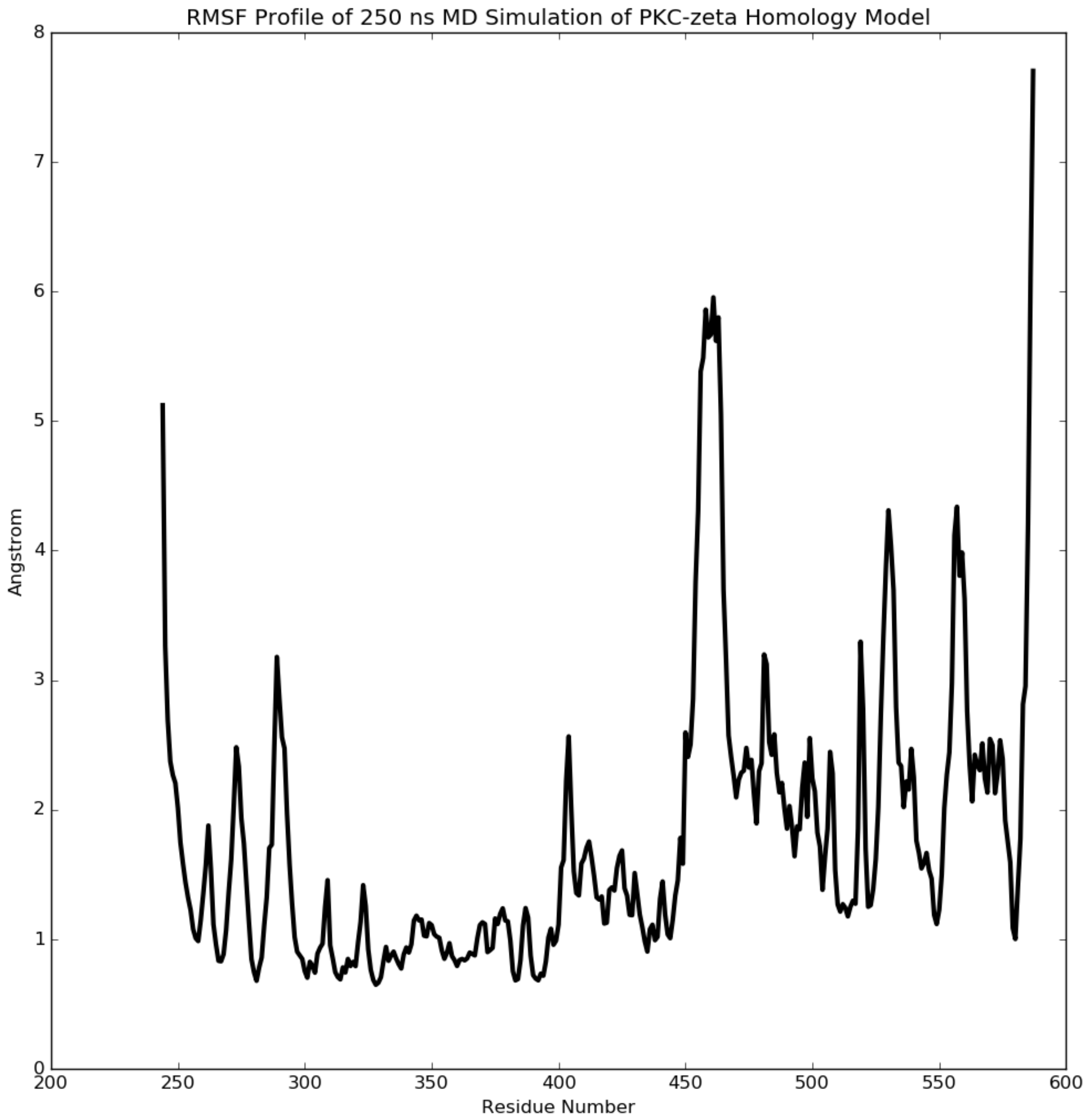

Supplemental Figure 4.) RMSF (Root Mean Square Fluctuation) plot for PKCζ Molecular Dynamics simulation. Graph shows RMSD per residue of α-carbon over length of simulation. The ATP region, approximately residues 300 – 450, show displacements under 2 Å. Most homologous residues show RMSD values well above 2 Å.
